# Borylated Monosaccharide 3-Boronic-3-deoxy-d-galactose: Detailed NMR Spectroscopic Characterisation, and Method for Spectroscopic Analysis of Anomeric and Boron Equilibria

**DOI:** 10.3390/ijms252212396

**Published:** 2024-11-19

**Authors:** Michela Simone

**Affiliations:** Discipline of Chemistry, University of Newcastle, Callaghan, NSW 2308, Australia; michela_simone@yahoo.co.uk

**Keywords:** monosaccharide, boronic acid, boronate, boron, BNCT, glycosidase, Fsp^3^ index, NMR, mutarotation, borarotation

## Abstract

Drug leads with a high Fsp^3^ index are more likely to possess desirable properties for progression in the drug development pipeline. This paper describes the first detailed NMR analysis of the borylated intermediate 3-deoxy-3-boronodiethanolamine-1,2:5,6-di-*O*-isopropylidene-α-d-galactofuranose and of the corresponding free monosaccharide analogue 3-boronic-3-deoxy-d-galactose in the early stage of the concurrent equilibrium processes of mutarotation and borarotation. A discussion of all potential equilibria is also presented alongside a comparison with relevant ^11^B-NMR data available from the scientific literature and our own library.

## 1. Introduction

**Figure 1:**
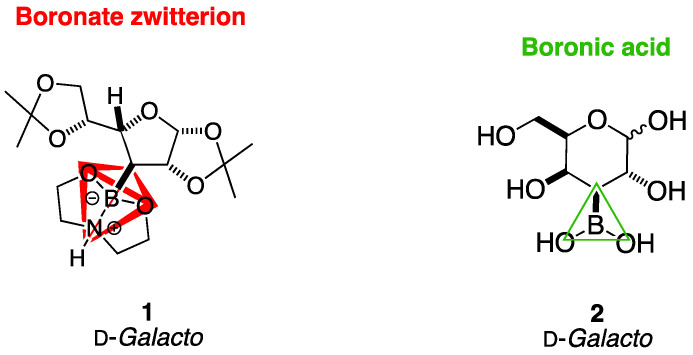
Structures of borylated intermediate **1** and borylated target molecule **2**, both possessing d-*galacto* absolute stereochemical configuration. Highlighted in red in **1**, the geometry of the B atom is tetrahedral, forming a zwitterion with the DEA. Highlighted via the green triangle in **2** is the B atom trigonal planar geometry.

The Lewis acidity of organic boron—coupled with its low intrinsic toxicity—make drug leads containing B pharmacophores highly desirable and of paramount importance in medicinal chemistry, through the expansion of the medicinal chemistry space and through the creation of new avenues for cancer management [1,2,3,4] and glycosidase inhibition [5,6,7,8,9,10,11,12,13,14]. The organic boron atom can favourably interact with enzyme active sites via the formation of reversible covalent bonds with nearby nucleophilic atoms. This expands the range of interactions between drugs and enzyme active sites to also include changes in the 3D shape and charge of a chemical group (e.g., trigonal -B(OH)_2_, tetrahedral -B^−^(OH)_3_, see Figure 1 as an example) and the range of applications to include BNCT and radiotherapeutic agents for boron neutron capture therapy (BNCT) that are more selective for cancer versus healthy cells, whose activation is wholly within clinical control, and that possess significantly reduced toxicity compared to more traditional chemotherapeutic agents.

The kaleidoscopic chemistry of organic boron, Its chemical behaviour, especially in relation to its Lewis acidity, and its pharmacophoric properties—as relating to boronic acid (R-B(OH)_2_) and boronate species (R-B(OR’)(OH), R-B(OR’)_2_, R-B^−^(OH)_3_, and R-B^−^(OR’)_3_)—are important in the development of a synthetic methodology to install organic B atoms on high Fsp^3^ index scaffolds and in the delineation of the equilibria in which the B atom becomes involved [5,6,15,16,17].

After communicating the expedited, robust, green, and highest yielding protocol for the installation of boronic acids on high Fsp^3^ index substrates, such as monosaccharides and their derivatives [18], in this paper, I present the first detailed NMR characterisation of borylated intermediate 3-deoxy-3-boronodiethanolamine-1,2:5,6-di-*O*-isopropylidene-α-d-galactofuranose **1** and of the corresponding free monosaccharide analogue 3-boronic-3-deoxy-d-galactose **2** (Figure 1). I also provide a method for the NMR spectroscopic analysis of the concurrent mutarotation and borarotation equilibria in aqueous solutions, supported by a discussion around all possible equilibria to consider when working with borylated monosaccharides, such as **2**. This paper provides the methodology for the NMR study of these complex equilibria and opens up an avenue for the comprehensive utilisation of borylated monosaccharides in BNCT and glycosidase modulation.

d-galactose was chosen as the starting material as it is involved in a panoply of biological functions, including cellular metabolism, and is a component of myelin in the brain and nervous system. Its biological functions seem quite well described along with disorders linked to galactose metabolism [19].

Intermediate **1** is a d-galactose analogue possessing a tetrahedral B atom bonded to a diethanolamine (DEA) protecting group which exists as a zwitterionic species. Target molecule **2** is also a d-galactose analogue, a 3-deoxy free monosaccharide possessing a trigonal planar B atom in the form of a boronic acid at C-3.

## 2. Results and Discussion

**Figure 2:**
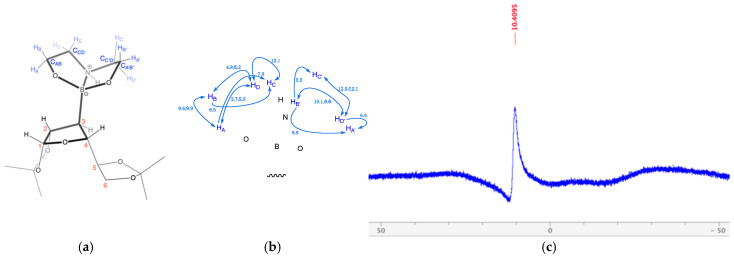
(**a**) Three-dimensional representation of zwitterion **1**, showing the numbering of the H and C atoms of the DEA group. (**b**) Highlighted in bold are the bonds of the THF ring, the C-B bond, and the DEA group (right). *J* couplings (Hz) were identified among the DEA H atoms. (**c**) ^11^B-NMR (128 MHz, CD_3_OD) of 3-deoxy-3-boronodiethanolamine-1,2:5,6-di-*O*-isopropylidene-α-d-galactofuranose **1** with labelled signal.

This section deals with the NMR analysis of 3-deoxy-3-boronodiethanolamine-1,2:5,6-di-*O*-isopropylidene-α-d-galactofuranose **1**. In ^11^B-NMR, the tetrahedral B signals generally appear in the range of 15–10 ppm [20,21,22,23,24,25,26]. The formation of the B-C bond in zwitterion **1** (Figure 2a) was confirmed by the appearance of a signal in the ^11^B-NMR at 10.4 ppm, indicating the presence of the tetrahedral B (Figure 2c) through the appearance of the broad C-3 signal at 37.9–35.2 ppm in the ^13^C-NMR, the shielded hydrogen H-3 in the ^1^H-NMR at 1.16 ppm (Figure S1), and the DEA group signals at 3.98–3.70 ppm, 3.35–3.17 ppm, and 3.00–2.85 ppm (Figure S1).

The DEA ^1^H-NMR signals were expected to appear as a result of two chemical shifts: one chemical shift for the two CH_2_ groups next to the O atoms and one chemical shift for the two CH_2_ groups next to the N atom (Figure 2b). Instead, eight signals were identified in the ^1^H-NMR spectrum and correlated through 2D NMR experiments (COSY, HSQC, and HMBC, Figures S5–S7). This indicates that there was hindered rotation about the B-C bond at NMR timescales and that, additionally, the N→B dative bond effectively locked the DEA group in a conformation, so that each of the four H atoms on each DEA arm produced its own chemical shift. Hence, each hydrogen gave rise to a signal (i.e., H_A_, H_A’_, H_B_, H_B’_, H_C_, H_C’_, H_D_, and H_D’_) (Figure 2 and Figure S1). Furthermore, the C atoms on each arm of the DEA appeared as a result of very close, but not identical, chemical shifts (i.e., C_A’B’_ at 63.8 ppm, C_AB_ is at 63.7 ppm, C_C’D’_ at 52.7 ppm, C_CD_ at 52.5 ppm), with four signals appearing instead of the expected two.

In Figure 2, the numbering system used for structure **1** (a) and the principal *J* couplings identified between the DEA H atoms (b) are highlighted.

H_A_, H_A’_, H_B_, and H_B’_ were more deshielded than H_C_, H_C’_, H_D_, and H_D’_. They were found at 3.94–3.88 ppm as a multiplet assigned to H_A’_, at 3.91 ppm as a dd assigned to H_B’,_ at 3.83 ppm as a ddd assigned to H_A_, and at 3.75 ppm as a ddd assigned to H_B_ (Figure S1). As it can be seen in Figure 2a,b, H_A_ was correlated with H_B_ (9.6 Hz), H_C_ (7.0 Hz), and H_D_ (2.7 Hz). H_B_ was correlated with H_A_ (9.9 Hz), H_C_ (6.5 Hz), and H_D_ (4.8 Hz). H_B’_ was correlated with H_A’_ and H_D’_ (9.9 Hz) and H_C’_ (5.5 Hz).

H_C_, H_C’_, H_D_, and H_D’_ were located at 3.28 ppm as a ddd assigned to H_C_, at 3.21 ppm as a ddd assigned to H_D’_, at 2.94 ppm as a ddd assigned to H_D_, and at 2.90 ppm as a dt assigned to H_C’_. The *J* coupling values for these were as follows and they mirrored their H_A/B_ counterparts discussed in the previous paragraph. H_C_ was correlated with H_D_ (12.0 Hz), H_A_ (7.6 Hz), and H_B_ (6.7 Hz). H_D’_ was correlated with H_C’_ (12.0 Hz), H_B’_ (10.1 Hz), and H_A’_ (6.9 Hz). H_D_ was correlated with H_C_ (12.1 Hz), H_B_ (5.2 Hz), and H_A_ (2.5 Hz). H_C’_ was correlated with H_D’_ (12.1 Hz).

The presence of a sharp signal at 10.4 ppm in the ^11^B-NMR spectrum indicates that the B atom was present in tetrahedral form and, thus, electronically shielded (Figure 2c).

This paragraph deals with the NMR analyses relating to the deprotection step. The NMR analysis of the synthesised borylated compounds revealed the appearance of more than one signal in the ^11^B-NMR spectra. ^11^B-NMR studies in the literature [27,28,29,30] are very limited, and, as such, the novel boron-containing entities reported here, in Refs. [5,6], provide further insights into the intramolecular (with nucleophilic atoms) and intermolecular (e.g., with solvent molecules) interactions of B atoms in high Fsp^3^ and low Fsp^3^ index compounds. A potentially wide range of reversible covalent interactions can be ascribed to the empty *p*-orbital of boron, an excellent Lewis acid.

This paragraph deals with the intramolecular and intermolecular B-O interactions, alongside the process of borarotation to equilibrium composition. The mutarotation equilibrium composition of the reference compound d-galactose in water is 30% α-pyranose, 64% β-pyranose, 2.5% α-furanose, 3.5% β-furanose, and 0.02% open-chain [31,32]. The target compound **2** was hypothesised to also prefer the pyranose anomeric conformations. In target compound **2**, two signals were observed at 31.7 ppm and 19.4 ppm (Table 1). The signal at 31.7 ppm corresponded to the trigonal planar boronic acid, while the signal at 19.4 ppm was hypothesised to have arisen from an interaction between the B atom and one of the nearby OH groups (Scheme S2), giving a tetrahedral boronate anion species such as **2B**, or as a result of complexation with a water molecule to give structures **3** and **4**. Such intramolecular interactions when the target compound **2** is in the furanose form do not seem possible for the d-galactose absolute stereochemical configuration, but may be possible for other monosaccharide configurations (Scheme S2, indigo box). Similar intramolecular interactions have been observed between B and O atoms, where the oxygen belongs to an ether (^11^B-NMR chemical shifts at 40 ppm) [33] or a carbonyl (^11^B-NMR chemical shifts at 16–14 ppm) [34,35].

Scheme S1 and Figure S2 show the postulated borarotation process for **1**.

### 2.1. NMR Analysis of the Target Compound 3-Boronic-3-deoxy-d-galactose ***2***

**Figure 3:**
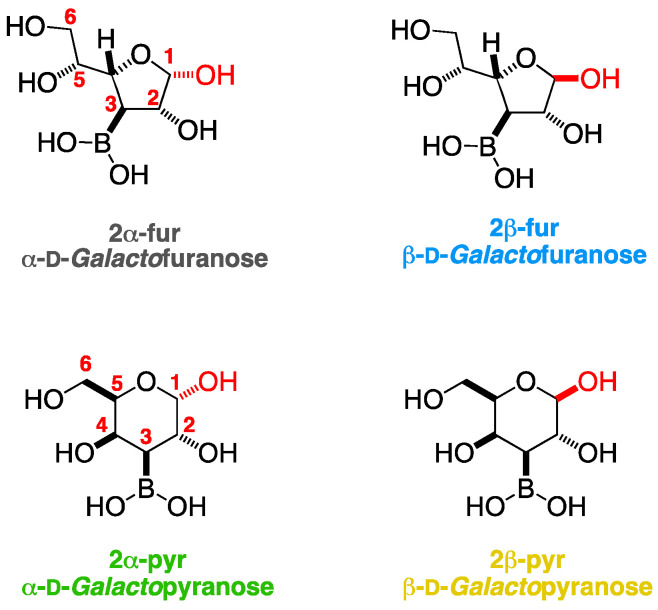
Structures of the four boronic acid anomers of the target compound **2**.

**Figure 4:**
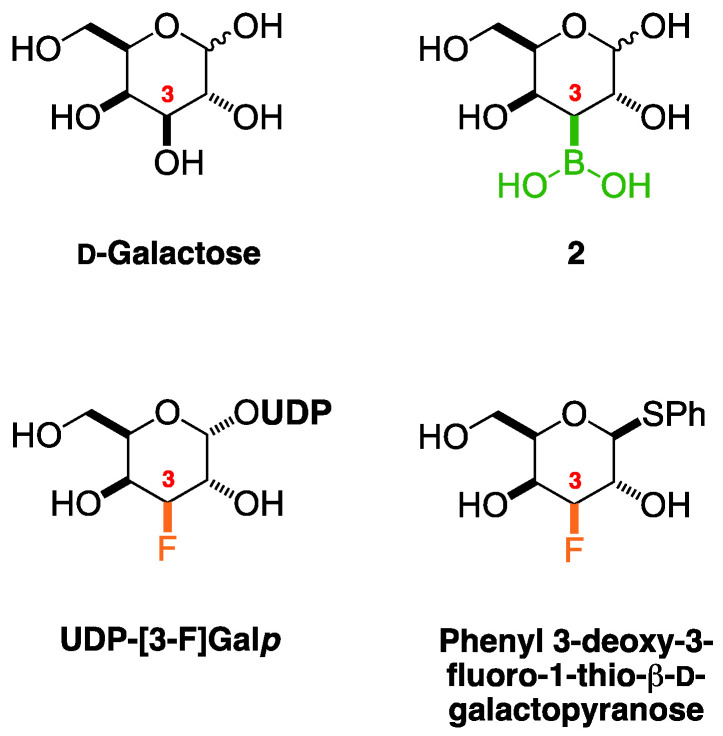
Structures of the target compound **2** and d-galactose in the pyranose forms, as well as of the UDP-[3-F]Gal*p* and phenyl 3-deoxy-3-fluoro-1-thio-β-d-galactopyranose for NMR data comparison.

**Figure 5:**
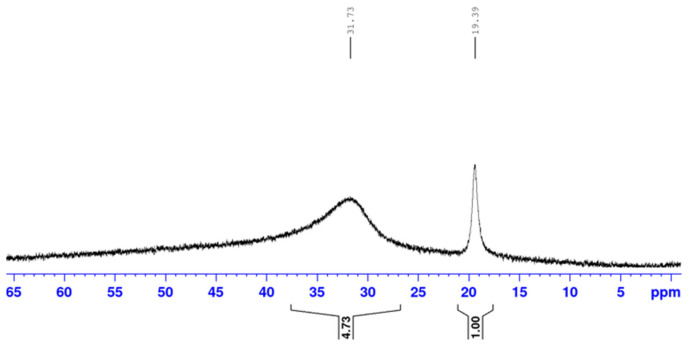
^11^B-NMR spectrum (128 MHz, D_2_O) of the target compound 3-boronic-3-deoxy-d-galactose **2**. The trigonal planar boronic acid species are present at 31.7 ppm and the slightly quarternised boronate ester species are present at 19.4 ppm.

The NMR spectra for the target compound **2** (Figure 3, Figure 4, Figure 5, Figures S3, S4, S8 and S9 and Table S2) were complex for two main reasons:

1. This molecule is expected to present as a mixture of anomers once mutarotation starts. Mutarotation is expected to start as soon as the acetonide protecting group between O-1 and O-2 is hydrolysed. Furthermore, it is not known how long the mutarotation would take to provide the equilibrium composition (Scheme S2, indigo box). However, it is advantageous to gather NMR datasets before mutarotation occurs, as the spectra are expected to be more easily identifiable as the various equilibrium species emerge.
2. The presence of a boronic acid group is expected to engage in equilibria with nucleophilic atoms (e.g., oxygens), both intramolecularly and intermolecularly, to potentially give a plethora of boronated species (namely boronic acids and boronates) through a process of borarotation. These equilibria deserve a separate study.

The spectra were run in D_2_O at 400 MHz as soon as the brown solid product was isolated (Supplementary Information, Figure S3). It was expected that these spectra would provide a snapshot into the beginning of the mutarotation and borarotation process.

The NMR spectroscopic literature on d-galactose and on the relevant derivatives substituted at the C-3 position [36,37] were studied in detail in order to provide insights into which anomers were present in the NMR tube at the time of analysis (see Table S2 for a summary and a comparison). Detailed NMR studies on d-galactose are available for the identification of signals in the ^13^C-NMR. Comprehensive ^1^H-NMR analyses for d-galactose hydrogens are not available in the chemical literature (e.g., the locations of furanose chemical shifts are incomplete).

From the NMR studies on d-galactose, it was possible to ascertain the anomeric composition for the target compound **2**, where the relative abundance of the anomeric forms was calculated by relating the magnitudes of integrations of the H-1 signals. These were the most isolated set of signals in the ^1^H-NMR spectrum. The anomeric composition was as follows: **2α-pyr**:**2β-pyr**:**2α-fur**:**2β-fur** ~0.25:1:1:1.

It also seems that signals from other minor species were starting to emerge (e.g., app triplet at 5.91 ppm, dd at 4.20 ppm, triplet at 1.78 ppm). These signals may have arisen from boronate species such as **3** and **4** (Scheme S2), as a result of the intermolecular addition of a water molecule to the empty *p*-orbital of the B atom. Intramolecular interactions between hydroxyl groups of **2** with the empty *p*-orbital of the B atom are likely to occur only when the target compound **2** is in the open-chain form. When **2** is in the pyranose and furanose forms, due to geometric constraints, the establishment of intramolecular bonds is unlikely. On the other hand, in the open-chain form, the lone pair of hydroxyls on OH-5 or OH-6 can form, respectively, furanose **5** and pyranose **6**, either found as the boric acid derivative or the boronate ester derivative (thus bearing a formal negative charge on the B atom). The compounds **5** and **6** possess, respectively, the d-*xylo*furanose and d-*xylo*pyranose absolute stereochemical configurations across the carbons C-3, C-4, and C-5 (see red numbering). Furthermore, it is thought that the compounds **5** and **6** are found as boronic acids (not as boronate esters), as the empty *p*-orbital on the B atom can accept the lone pair of either oxygen bound to it in such a way as to establish an anomeric-type equilibrium at position B (analogous to C-1 in free monosaccharides).

The comparison of the target compound 2 to the parent compound d-galactose [38,39,40,41,42,43,44] and to d-galactose derivatives possessing an electronegative substituent at C-3 [36,37] was appropriate in order to analyse the effect of substitution at the C-3 position. To the best of my knowledge, NMR data for 3-deoxy-3-fluoro-d-galactose were not available at the time of this study; therefore, phenyl 3-deoxy-3-fluoro-1-thio-β-d-galactopyranoside [36] and UDP-[3-F]Gal*p* [37] were considered instead. The presence of an electron-withdrawing group, such as fluorine, shifted H-3 from 3.65 ppm in d-galactose (β-pyr) to 4.60 ppm for phenyl 3-deoxy-3-fluoro-1-thio-β-d-galactopyranoside and to 4.84 ppm in UDP-[3-F]Gal*p*. The C-3 signal in the ^13^C-NMR also shifted from 73.8 ppm to 93.9 ppm and 92.5 ppm, respectively. On the other hand, the presence of an atom that is less electronegative than both carbon and hydrogen, such as boron, produced upfield shifts compared to d-galactose, namely to 1.47 ppm with respect to H-3 and to 31.3–32.3 ppm with respect to C-3 of the target compound **2**. The presence of other atoms (e.g., F, B) bonded to C-3, in place of the hydroxyl group, did not have any significant impact on the chemical shifts of hydrogens and carbons beyond H-3 and C-3, via the σ-system of electrons.

The NMR data for the parent compound d-galactose [38,39,40,41,42,43,44] seem incomplete, in particular in terms of the furanose hydrogen signals missing. Only in [42] the location of the chemical shift for the H-1s was surmised from available, albeit fragmented, data. Here, the chemical shifts for all the hydrogens and carbons for both furanose forms of the target compound **2** are fully characterised. The location of the two H-1s of compound **7** (5.25 ppm and 5.27 ppm for α-fur and β-fur, respectively) were found to be in line with the locations of H-1s with respect to the parent compound (~5.23–5.27 ppm).

### 2.2. General Observations for the ^13^C-NMR Spectrum of 3-Boronic-3-deoxy-d-galactose ***2***

The following considerations are relevant for the target compound **2**, through analysis of the ^13^C-NMR data of its parent compound d-galactose:

1. Carbons C-2, C-3, C-4, and C-5 for both (α- and β-) furanose forms are found in the 71–83 ppm range (in D_2_O) [40,43]. This is a significantly different range compared to that of the same carbons for the pyranose forms which are found in the 69–76 ppm range. This range difference can be useful in the analysis of anomeric mixtures, in guiding an initial speculation about anomeric composition.
2. Carbon locations that do not change across the pyranose and furanose forms are the ones for C-6, which are found narrowly in the 62–63 ppm chemical shift range. This information is useful if coupled with the DEPT spectrum. There is only one CH_2_ group, and the number of signals in this range provides insights into how many anomeric species are present. On the other hand, it could be difficult to determine which signal belongs to which anomeric species if these signals are so close to one another.
3. The locations of C-1 signals can be the most useful piece of information to identify which anomeric species are present. The α-pyranose C-1 is found at 93 ppm, the β-pyranose and α-furanose C-1s are found at 96–97 ppm, and the β-furanose C-1 is located at 102 ppm.
4. The C-3 signals are expected to be broadened due to the presence of the quadrupolar B nuclei one bond away.

The next section should be read in conjunction with the chemical shift data summarised in Table S2 and with the NMR spectra in Figure 4, Figure 5, Figure S3 (^1^H), Figure S4 (^13^C), Figure S8 (COSY) and Figure S9 (HSQC), respectively and provides elucidation of the predominant four spin systems present for the target compound **2**.

This section deals with the **β-furanose anomer (indigo)**. Analysis of the NMR data for the target compound **2** started from the ^13^C-NMR, where a signal at 102.2 ppm stood out and was identified. This was identified as the C-1 carbon for the β-furanose (β-fur) form. The HSQC correlation (Figure S9) of this signal with a doublet (d) at 5.27 ppm in the ^1^H-NMR allowed for the localization of the corresponding H-1 (β-fur). The COSY correlation (Figure S8) of this H-1 with a doublet of doublet (dd) at 4.27 ppm revealed the location of H-2 (β-fur). The COSY correlation of this H-2 with a dd at 1.68 ppm revealed the location of H-3 (β-fur). This signal was partially obscured by another H-3. The COSY correlation of this H-3 with a dd at 4.40 ppm revealed the location of H-4 (β-fur). HMBC correlations allowed for the localization of H-5 (β-fur) at ~3.70 ppm; however, the exact location could not be established as the area of the ^1^H-NMR spectrum between 3.50 ppm and 3.75 ppm contained many overlapping signals. Similarly, 2D NMR correlations from the approximate location of H-5 (β-fur) allowed for the localization of H-6 and H-6’ (β-fur) in the range of 3.60–3.75 ppm. Again, in this case as well, due to the plethora of overlapping signals, it was not possible to locate H-6 and H-6’ (β-fur) accurately.

HSQC correlations from the identified H signals back to the ^13^C-NMR spectrum revealed the locations of C-2 (β-fur) at 78.7 ppm, C-3 (β-fur) as a broad signal at 35.0–36.8 ppm, C-4 (β-fur) at 80.2 ppm, C-5 (β-fur) at 73.9 ppm (tentative assignment), and C-6 (β-fur) at either 62.9 ppm or 62.7 ppm. The locations of these C signals were around the location of the same carbons for d-galactose. The main difference was the location of the C-3 in 2 which was found to be present as a broad signal between 35.0 ppm and 36.8 ppm, reflecting the shielding effect that the nearby boron had on C-3 and the coupling effect which broadened the C-3 signal. C-2 and C-4 of 2 appeared in slightly higher fields. These minor shifts were probably due to the shielding effect the boron transmits to C-2 and C-4 via the σ-system of the electrons.

The *J* coupling values for the hydrogens (β-fur) were as follows: *J*_H-1,H-2_ 2.3 Hz, *J*_H-2,H-3_ 5.6 Hz, *J*_H-3,H-4_ 7.9 Hz, and *J*_H-4,H-5_ 4.1 Hz. *J*_H-5,H-6_ and *J*_H-6,H-6’_ could not be reliably identified due to the many overlapping signals.

This paragraph deals with the **β-pyranose anomer (yellow)**. The doublet at 4.62 ppm could belong to the H-1 of the β-pyranose (β-pyr) form. It was correlated (COSY) with a partially obscured dd centred around 3.79 ppm which could be H-2 (β-pyr). This H-2 was correlated with a dd centred around 1.47 ppm which could be H-3 (β-pyr). This signal correlated with a partially obscured broad singlet at 4.08–4.12 ppm which could be H-4 (β-pyr). This signal was not COSY correlated with any other hydrogen. H-5, H-6, and H-6’ could not be reliably located in the chemical shift range of 3.60–3.85 ppm due to the many overlapping signals. However, H-6 and H-6’ should be in the range of 3.73–3.81 ppm. This determination was allowed by the slightly broad signal appearing in the DEPT for a small carbon signal of the CH_2_-type at 61.3 ppm. This signal was reminiscent of the broadened carbon signals identified for this anomeric form. As a general consideration, the location of the signals was in line with the locations expected for this anomeric form in d-galactose, with the difference that H-3 for 2 was significantly more shielded as a result of the nearby boron.

HSQC correlations allowed for the identification of the carbon signals in the β-pyr anomeric form. C-1 was present as a slightly broadened signal (15 Hz wide) at 98.6 ppm, C-2 as a broadened signal (14 Hz) at 68.0 ppm, C-3 as a broad signal at 31.3–32.3 ppm, and C-4 as a broadened signal (71 Hz) at 67.0–67.7 ppm. C-5 was classified as a broadened signal at 79.9–79.3 ppm (62 Hz) via an HSQC correlation with a signal centred around 3.78 ppm in the ^1^H-NMR spectrum. C-6 was found to occur at 61.3 ppm as a broadened signal (18.5 Hz), appearing in the DEPT spectrum on the same side as the C-6 CH_2_ signals (62.9 and 62.7 ppm). The presence of so many broadened signals in the ^13^C-NMR for the β-pyr (and also the α-pyr) anomer points to a possible interaction between the boronic acid group and the monosaccharide hydroxyls, on one hand, and the structured component of water [32], on the other, slowing down the conformational movements of the β-pyr form closer to the NMR timescales.

This also logically points to the postulate that the α-pyr form also experiences a broadening of the C-signals. However, since a significantly smaller percentage composition of the α-pyr form was present, then it was expected that such broadened signals may hide in the baseline noise.

The *J* coupling values for the hydrogens (β-pyr) were as follows: *J*_H-1,H-2_ 7.8 Hz, *J*_H-2,H-3_ 11.4 Hz, and *J*_H-3,H-4_ 2.6 Hz. *J*_H-4,H-5_, *J*_H-5,H-6_, and *J*_H-6,H-6’_ could not be identified due to the many overlapping signals and the broad singlet for H-4. The *J* coupling values match the magnitudes expected for this type of correlation in this anomeric form [42].

This paragraph deals with the **α-furanose anomer (grey)**. For the α-furanose (α-fur) form, the signal at 94.4 ppm was assigned to C-1. The HSQC correlation between C-1 and the doublet centred around 5.25 ppm was classified as H-1 (α-fur). COSY correlations allowed to identify the hydrogens in the same spin system, where H-2 was a dd at 4.31 ppm, H-3 was a triplet at 1.68 ppm, and H-4 was a dd at 4.13 ppm. H-5 was tentatively classified as a signal centred around 3.75 ppm as a result of the many overlapping signals in this region. H-6 and H-6’ were tentatively assigned to the 3.60–3.75 ppm range, overlapping with many other signals. The only useful information in the latter case was obtained from the HSQC, which showed a correlation between the ^1^H-NMR 3.60–3.75 ppm range and the two C-6 signals in the ^13^C-NMR appearing at 62.9 ppm and 62.7 ppm.

HSQC correlations also correlated H-2 with a carbon at 74.7 ppm which was classified as C-2 (α-fur, 14 Hz), H-3 with a broad signal at 31.3–32.3 ppm classified as C-3 (α-fur), H-4 with a signal at 80.6 ppm classified as C-4 (α-fur), and—tentatively—H-5 with a signal at 73.1 ppm classified as C-5 (α-fur).

The locations of these C signals were similar to the locations of the same carbons with respect to d-galactose. The main difference was the location of C-3 in 2, C3 being a broad signal between 31.3 ppm and 32.3 ppm, reflecting the shielding effect that the nearby boron had on C-3 and the coupling effect which broadened the C-3 signal. C-2 and C-4 occurred in a slightly higher field. These minor shifts were probably due to the shielding effect that the boron transmitted to C-2 and C-4 via the σ-system of the electrons.

The *J* coupling values for the hydrogens (α-fur) were as follows: *J*_H-1,H-2_ 4.6 Hz, *J*_H-2,H-3_ 11.3 Hz, *J*_H-3,H-4_ 10.8 Hz, and *J*_H-4,H-5_ 5.2 Hz. *J*_H-5,H-6_ and *J*_H-6,H-6’_ could not be identified due to the many overlapping signals.

This paragraph deals with the **α-pyranose anomer (green)**. The fourth anomeric form, the α-pyranose (α-pyr), was present as a minor component in this NMR snapshot. The H-1 (α-pyr) occurred as a partially obscured d at 5.24 ppm. A COSY correlation at 4.14 ppm indicates that the H-2 (α-pyr) signal was hidden under the H-4 (α-fur). From here, it was not possible to utilize COSY correlations to identify the remainder of the hydrogens in this spin system.

The location of H-3 (α-pyr) is thought to have been hidden at 1.43–1.50 ppm under H-3 (β-pyr) because of the structural similarities between the two pyr forms, as H-3s for both fur forms appeared at 164–172 ppm. The HSQC correlation of H-1 with a signal at 91.2 ppm in the ^13^C-NMR established that this was C-1 (α-pyr). An analysis of the ^13^C-NMR revealed a number of still unassigned signals at 71.9 ppm and a broad signal at 70.4–71.4 ppm (104 Hz), 65.0 ppm (12 Hz), and 64.8 ppm. The signal at 65.0 ppm was classified as C-2 (α-pyr), as it exhibited an HSQC correlation at 4.16 ppm in the ^1^H-NMR which is where the H-2 (α-pyr) was hidden. It was a slightly broadened signal, in line with other slightly broadened C-2 signals (e.g., C-2 (α-fur, 14 Hz) and C-2 (β-pyr, 14)). The signals at 71.9 ppm and 70.4–71.4 ppm were classified as C-5 and C-4 (α-pyr), respectively, in line with d-galactose signal locations, chemical shift locations of the same carbons in the other anomeric forms analysed, and signal shape (C-4 was broad as the other pyr C-4 (β-pyr, 71 Hz), while C-5 was a sharp signal as most other C-5s). C-3 was expected to be so broad and shallow that it would be lost in the chemical shift scale. It could not be located. The signal at 64.8 ppm was HSQC correlated with two signals in the ^1^H-NMR spectrum centred around 3.69 ppm and 3.61 ppm. This revealed the locations of C-6, H-6, and H-6’ (α-pyr).

The HSQC correlation between the broad C-4 (α-pyr) and the broad singlet, partially obscured, at 4.06–4.14 ppm, allowed for the classification of this singlet as H-4 (α-pyr). H-5 was embedded in the region of 3.60–3.85 ppm; however, no correlations were discernible. Tentative assignments were made based on d-galactose anomeric form assignments, the remaining unassigned integrations/signals, and through the analysis of the other NMR spectra.

The *J* coupling values for the hydrogens (α-pyr) were not discernible due to most of these signals being either partially obscured or completely buried under the signals of the three other predominant forms.

In the ^11^B-NMR spectrum (Figure 5), it is possible to see two signals:

- The broad one at 31.7 ppm which encompasses the trigonal planar boronic acid species. This signal is likely (further) broadened by the quadrupolar ^11^B spin and possesses a lower symmetry.
- The relatively sharper signal at 19.4 ppm which corresponds to slightly quaternised boronate ester species, less broadened by the quadrupolar ^11^B spin and possessing higher symmetry.

The latter can arise from intramolecular (e.g., -OH) and/or intermolecular (e.g., H_2_O) nucleophilic species datively donating an electron pair to the boron empty *p*-orbital. The integrations of these two signals show that the trigonal planar geometry was the predominant form in which the borons were found, most likely as boronic acids. Due to the broadening of the signal at 31.7 ppm, the background signal arose from the NMR tube [45], and, due to the spectra showing an early snapshot in time of the aqueous solution of **2** that may have not yet reached mutarotation and equilibration of the boron species (borarotation), the integration ratio was indicative of the boron species present.

## 3. Materials and Methods

This section deals with nuclear magnetic resonance (NMR). One-dimensional and two-dimensional spectra were recorded on a Bruker Ascend^TM^ 400 (Bruker Daltonics GmbH & Co. KG, Bremen, Germany) in the deuterated solvent stated. Chemical shifts (δ) were expressed in ppm and the coupling constants (*J*) in Hz. Residual signals from the deuterated methanol (3.31 ppm for ^1^H-NMR and 49.00 ppm for ^13^C-NMR) and deuterium oxide (4.89 ppm for ^1^H-NMR) were used as an internal reference [46]. The NMR spectra in the Supplementary Information were produced using TopSpin 4.2.0.

## 4. Conclusions

A detailed spectroscopic analysis of C-3 borylated d-galactose **2** and its DEA-protected precursor **1** was carried out. This paper—the first of its kind in the area of borylated monosaccharides—provides a methodology to carefully characterise these new classes of drug leads, principally by NMR, highlighting the challenges related to the concurrent mutarotation and borarotation equilibria in aqueous solutions. The spectra were run at the beginning of the mutarotation and borarotation processes to allow for the characterisation of the pyranose and furanose forms. Data for analogous systems were analysed for comparison purposes. This study provides a trampoline to further encourage the characterisation efforts related to these systems, with a view to studying the species occurring once mutarotation and borarotation have been reached via spectroscopy, spectrometry, and computational means.

## Figures and Tables

**Table 1:** ^11^B-NMR data for the intermediate **1** and the target compound **2** with the proposed assignment of the signals observed.

Compound	Solvent	Chemical Shifts (Integration Magnitude)	Geometry of B Atom	Proposed Assignment
**1** (Scheme S2)	MeOD	10.4 (1.00)	Tetrahedral	B makes four bonds (zwitterion)
**1** (in equilibrium) (Scheme S2)		30.9 (0.02)	Trigonal planar	B makes three bonds
		17.2 (0.03)	Partially tetrahedral	B makes a partial fourth bond
		10.3 (1.00)	Tetrahedral	B makes four bonds (zwitterion)
**2**	D_2_O	31.7 (4.73)	Trigonal planar	B makes three bonds (boronic acid)
		19.4 (1.00)	Partially tetrahedral	B makes a partial fourth bond via intramolecular and/or intermolecular interaction/s (boronates)

^11^B-NMR data were collected from a range of reference compounds (**9**–**11**) available in our inventory (Table S1) and from borylated systems recently investigated [5,6]. The aim is the development of a more comprehensive hypothesis in regard to the equilibria observed in the NMR spectra via, primarily, analysis and assignment of the observed ^11^B-NMR signals.

## Data Availability

The original contributions presented in the study are included in the article and Supplementary Materials, further inquiries can be directed to the corresponding author.

## Supplementary Materials

The following supporting information can be downloaded at: https://www.mdpi.com/article/10.3390/ijms252212396/s1. References [5,6,25,36,37,39,40,41,42,43,47,48,49] are cited in Supplementary Materials.
