# Supplementary material for: Borylated Monosaccharide 3-Boronic-3-deoxy-d-galactose: Detailed NMR Spectroscopic Characterisation, and Method for Spectroscopic Analysis of Anomeric and Boron Equilibria
Source: Int J Mol Sci. 2024 Nov 19;25(22):12396. doi: 10.3390/ijms252212396 (PMC11594630; doi:10.3390/ijms252212396)
Supplement: Supplementary file 1 [file ijms-25-12396-s001.zip › ijms-3303957-supplementary.pdf]

Supplementary Information

Borylated Monosaccharide 3-Boronic-3-deoxy-D-galactose: Detailed NMR Spectroscopic Characterisation, and Method for Spectroscopic Analysis of Anomeric and Boron Equilibria.

Michela Simone*[a,b]

[a]Discipline of Chemistry, University of Newcastle, Callaghan, NSW 2308, Australia. [b]*Currently at*: Newcastle CSIRO Energy Centre, 10 Murray Dwyer Circuit, Mayfield West, NSW2304, Australia.

E-mail: michela_simone@yahoo.co.uk

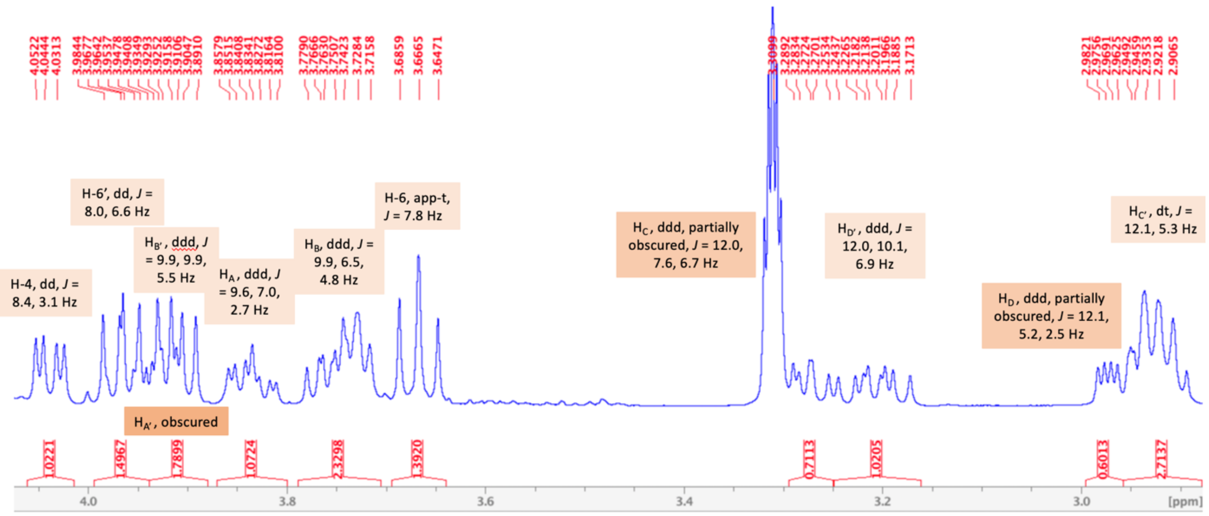

Figure S1. 4.08-2.87 ppm section of the ^{1}H-NMR (400 MHz, CD_3OD) of 3-deoxy-3-boronodiethanolamine-1,2:5,6-di-O-isopropylidene-α-D-galactofuranose **1** with labelled signals and integration magnitudes. Obscured and partially obscured signals are highlighted in darker shades of orange. Residual DEA at 3.75 ppm and 2.90 ppm.

Table S1. Structures of the reference compounds analysed by ^{11}B-NMR and their corresponding signals at 128 MHz, dissolved in the deuterated solvent stated in brackets and at neutral pH.

Structure	Chemical shift, ppm	Reference	Structure	Chemical shift, ppm	Reference
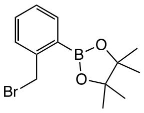 **ortho 9**	31 (D_2O)	Commercially available	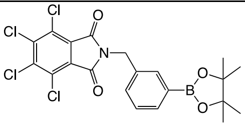 **meta 13**	31.5 ($CDCl_3$)	[1]
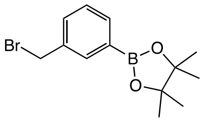 **meta 9**	31 (D_2O)	Commercially available	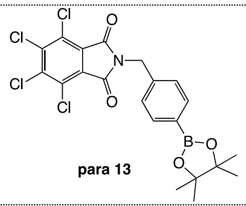 **para 13**	31.1 ($CDCl_3$)	[1]
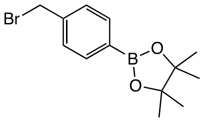 **para 9**	31 (D_2O)	Commercially available	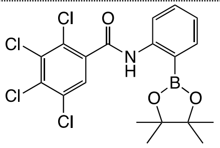 **ortho 14**	31.1 ($CDCl_3$)	[1]
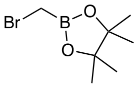 **10**	Rapidly decomposed (D_2O)	Commercially available	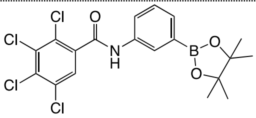 **meta 14**	31.7 ($CDCl_3$)	[1]
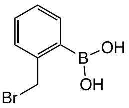 **ortho 11**	30 (D_2O)	Commercially available	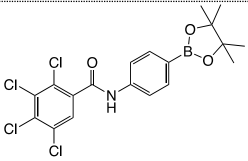 **para 14**	31.8 ($CDCl_3$)	[1]
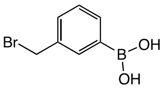 **meta 11**	29 (D_2O)	Commercially available	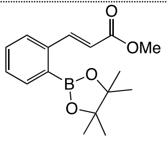 **ortho 15**	31.5 ($CDCl_3$)	[2]
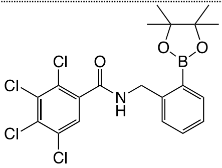 **ortho 12**	31.8 ($CDCl_3$)	[1]	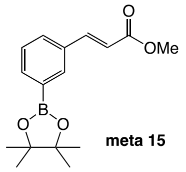 **meta 15**	30.7 ($CDCl_3$)	[2]
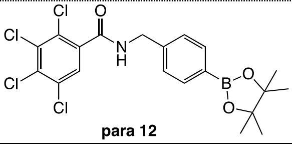 **para 12**	31.6 ($CDCl_3$)	[1]	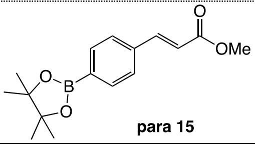 **para 15**	31.2 ($CDCl_3$)	[2]

Protected Trigonal Planar Boron Species. Most trigonal planar boron species (whether aliphatic or aromatic) have been found in ^{11}B-NMR within a range of 90-15 ppm depending on the substitution pattern.[3] For boronic esters and boronic acids this range is generally 31-18 ppm.[3,4] The aromatic trigonal planar boron esters (e.g. protected by the pinacol) of these compounds was generally found as a sharp peak at ≈32-30 ppm which was observed in **9**, **11**-**15** (Table S2).[1,2] In reference compounds **9** (*ortho*, *meta*, *para*) this boron ester is also observed at 31 ppm, in line with literature data.[5] Compound **10** decomposed rapidly in D_2O, whereas boronic acids **11** were found slightly upfield at 30-29 ppm.

Equilibria involving 3-deoxy-3-boronodiethanolamine-1,2:5,6-di-*O*-isopropylidene-α-D-galactofuranose 1

During the optimisation of this purification protocol, it was observed that the introduction of the trituration with diethyl ether resulted in the appearance of only one species in the ^{1}H-NMR spectra of the solid that remained undissolved, while the filtrate was found to contain unidentified compounds. By careful analysis of ^{11}B-NMR and ^{1}H-NMR spectra, these unidentified compounds were found to contain borylated monosaccharide species. It was hypothesised that these species are not impurities, but product **1** in equilibrium with the DEA group, where the B-O bonds momentarily break and reform. This also would facilitate rotation around the B-C bond (Scheme S1).

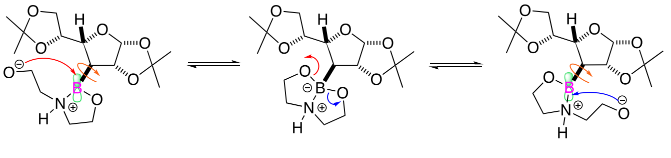

1
D-*Galacto*

Scheme S1. Hypothesised equilibrium occurring in intermediate **1**, with the B-O bonds temporarily breaking and reforming. Highlighted are the B atoms (purple when in a geometry approaching trigonal planarity), the empty *p*-orbital of the boron (in green) and the alternate breaking and reforming of either B-O bond (red arrows, blue arrows). The orange arrows show the rotation about the C-B bond.

This process can be seen clearly after a certain amount of time has elapsed (>24 hours). It is a speciation-type process which is analogous to the establishment of anomeric equilibria (mutarotation) and it has been designated as borarotation. This is not the only borarotation process that can be discerned. Target molecule **2** also goes through a borarotation process, which can be seen in the structures outside the indigo box in Scheme S2.

Analysis of the H-1 region (5.8-5.6 ppm) in the ^{1}H-NMR spectrum indicated the presence of four major species in a 1.00 (blue) : 0.51 (red) : 0.24 (yellow) : 0.15 (green) ratio (Figure S2). Three of these could be identified by ^{1}H- and ^{13}C-NMR (using COSY, HMBC and HSQC correlations). In the insert are shown the four H-1 peaks and their integration ratios.

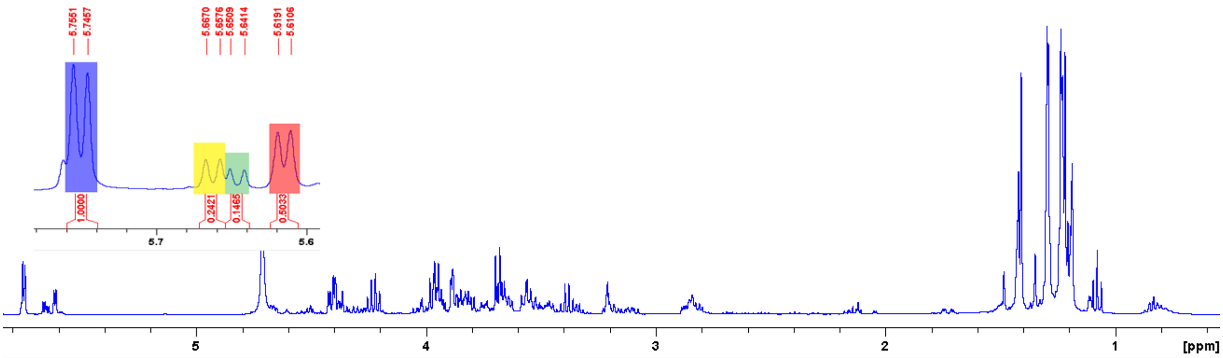

Figure S2. ^{1}H-NMR (400MHz, MeOD) of the diethyl ether filtrate from purification of **1** showing the hypothesised equilibrium species. The insert shows the signals in the H-1 region (5.8-5.6 ppm). Four main species (potentially five) can be seen in the ratio 1.00 (blue):0.51 (red):0.24 (yellow):0.15 (green). The structure shows the rotation around the B-C bond resulting from the hypothesised equilibrium.

Equilibria involving 3-boronic-3-deoxy-D-galactose 2

Scheme S2. Intramolecular and intermolecular interactions contributing to mutarotation and borarotation of target compound **2**.

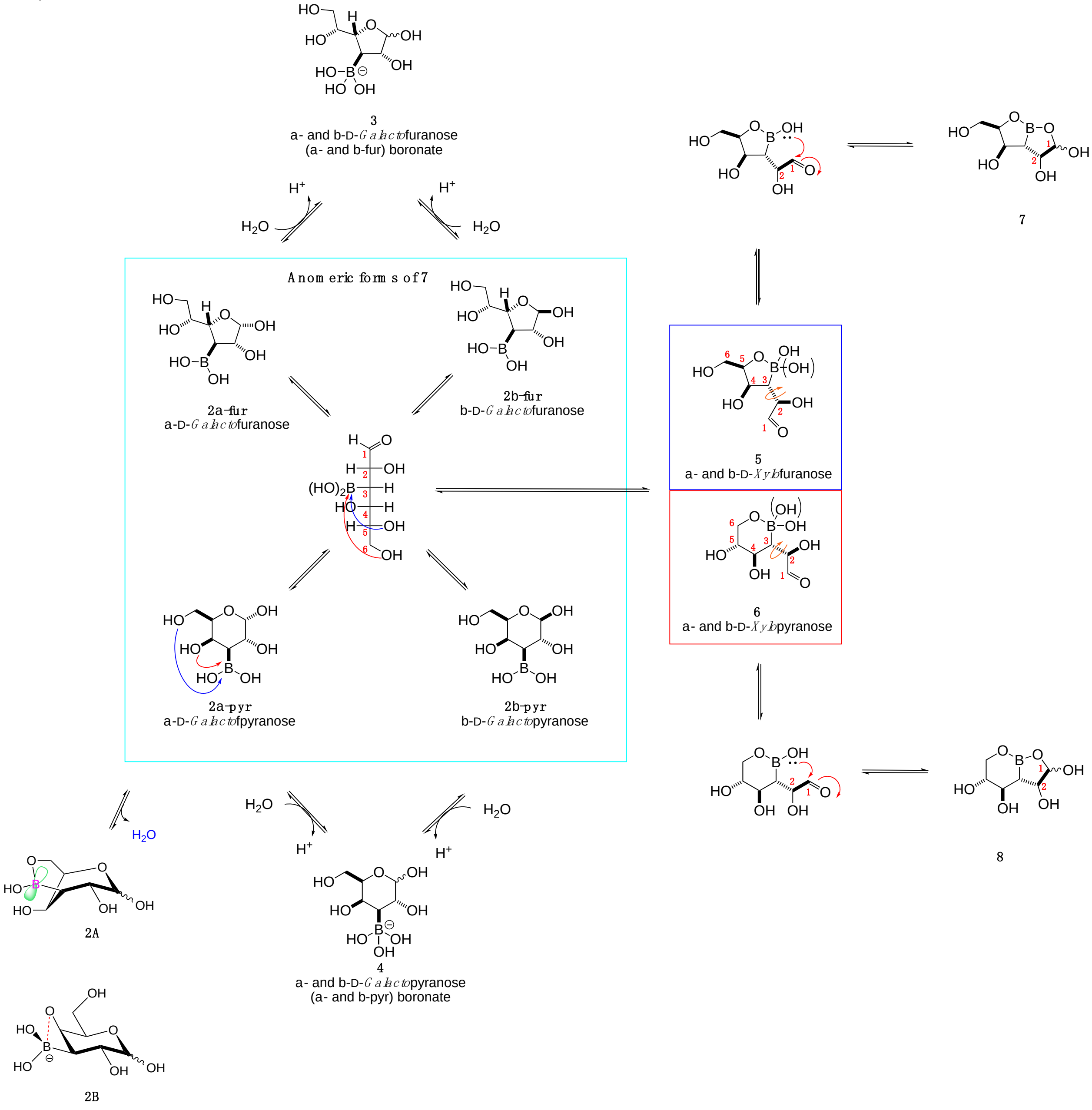

In the indigo box (Scheme S2) the anomeric forms of target compound **2** can be seen. In the blue box, compound **5** derives from the intramolecular bond (blue arrow) between OH-5 of **2** (open chain form) and the boron empty *p*-orbital. In the red box, compound **6** derives from the intramolecular bond (red arrow) between OH-6 of **2** (open-chain form) and the boron empty *p*-orbital. Equilibria established between B-OH and the aldehyde C=O of **5** provide the anomeric mixture **7**. Similarly, equilibria established between B-OH and the aldehyde C=O of **6** provide the anomeric mixture **8**. Hypothesised intramolecular interactions of the B atom of target compound **2** in the pyranose forms with nearby OH groups give rise to equilibrium structures **2A** and **2B**.

Table S2. Summary of chemical shifts for ^{1}H- and ^{13}C-NMR signals of anomeric forms of target compound **2**, and comparison to the chemical shifts for ^{1}H- and ^{13}C-NMR signals of anomeric forms of parent structure D-galactose. Comparison also with D-galactose derivatives, phenyl 3-deoxy-3-fluoro-1-thio-β-D-galactopyranose[6] and UDP-[3-F]Gal*p*,[7] containing a highly electronegative substituent at C-3 helps to discern the electronic effect of substituents on the D-galactose structure.

	α-pyranose (ppm)			β-pyranose (ppm)			α-furanose (ppm)		β-furanose (ppm)	
	**D-Galactose**	**Target compound 2**	**UDP-[3-F]Gal*p*[7]**	**D-Galactose**	**Target compound 2**	**Phenyl 3-deoxy-3-fluoro-1-thio-β-D-galacto-pyranose[6]**	**D-Galactose**	**Target compound 2**	**D-Galactose**	**Target compound 2**
**C-1**	93.2[8]<br>92.4[9]<br>93.2[10]	**91.2**	97.4, dd,<br>$J_{C-1,P}$ 11 Hz,<br>$J_{C-1,F}$ 7 Hz	96.5[9]<br>97.3[8,10]	**98.6**,<br>broadened, 15 Hz#	87.0, d,<br>$J_{C-1,F}$ 8.7 Hz	95.8[8,10]	**94.4**	101.8[10] [8]	**102.2**
**C-2**	69.4[8,9]	**65.0**	68.6, dd,<br>$J_{C-2,F}$ 19 Hz,<br>$J_{C-2,P}$ 9 Hz	72.9[8]<br>71.9[9]	**68.0**,<br>broadened, 14 Hz#	66.9, d,<br>$J_{C-2,F}$ 16.5 Hz	77.1[8]	**74.7**	82.2[8]	**78.7**
**C-3**	70.2[8]<br>68.4[9]	*Not resolved*	92.5, d,<br>$J_{C-3,F}$ 181 Hz	73.8[8]<br>72.9[9]	**32.3-31.3**,<br>broad	93.9, d,<br>$J_{C-3,F}$ 185 Hz	75.1[8]	**32.3-31.3**,<br>broad	76.6[8]	**36.8-35.0**,<br>broad
**C-4**	70.3[8]<br>69.2[9]	**71.4-70.4**, broadened,<br>104 Hz#	68.9, d,<br>$J_{C-4,F}$ 17 Hz	69.7[8]<br>68.8[9]	**67.7-67.0**, broadened,<br>71 Hz#	67.5, d,<br>$J_{C-4,F}$ 19.3 Hz	81.6[8]	**80.6**	82.8[8]	**80.2**
**C-5**	71.4[8]<br>70.5[9]	**71.9**	73.9, d,<br>$J_{C-5,F}$ 6 Hz	76.0[8]<br>75.2[9]	**79.9-79.3**,<br>broadened, 62 Hz#	77.9, d,<br>$J_{C-5,F}$ 6.5 Hz	*Not resolved*[8]	**73.1**	71.5[8]	**73.9***
**C-6**	62.2[8]<br>61.3[9]	**64.8**	62.2	62.0[8]<br>61.1[9]	**61.3**, broadened,<br>18.5 Hz#	60.5	63.3[8]	**62.9***	63.6[8]	**62.7***
**H-1**	5.23, d<br>$J_{H-1,H-2}$ 3 Hz[11]<br>5.34, d,<br>$J_{H-1,H-2}$ 2.8 Hz[12]<br>$J_{H-1,H-2}$ 3.2 Hz[11]	**5.24**, partially obscured	5.72, dt,<br>$J_{H-1,P}$ 7.0 Hz,<br>$J_{H-1,H-2}$ 3.5 Hz	4.55, d,<br>$J_{H-1,H-2}$ 7.2 Hz[13]<br>4.68, d,<br>$J_{H-1,H-2}$ 7.1 Hz[12]<br>$J_{H-1,H-2}$ 7.5 Hz[11]	**4.62**, d,<br>$J_{H-1,H-2}$ 7.8 Hz	4.76, d,<br>$J_{H-1,H-2}$ 10.0 Hz	~5.23-5.27[11]	**5.25**, d,<br>$J_{H-1,H-2}$ 4.6 Hz	~5.23-5.27[11]	**5.27**, d,<br>$J_{H-1,H-2}$ 2.3 Hz
**H-2**	3.87	**4.16-4.10**, obscured by overlapping signals	4.12, tt,<br>$J_{H-2,H-3}$ 8.5 Hz,<br>$J_{H-2,H-1}$ 3.5 Hz	3.49,<br>$J_{H-2,H-3}$ 10.5 Hz[11]	**3.79**, partially obscured, dd,<br>$J_{H-2,H-3}$ 11.4 Hz,<br>$J_{H-2,H-1}$ 7.9 Hz	3.81, m		**4.31**, dd,<br>$J_{H-2,H-3}$ 11.3 Hz,<br>$J_{H-2,H-1}$ 4.3 Hz		**4.27**, dd<br>$J_{H-2,H-3}$ 5.6 Hz,<br>$J_{H-2,H-1}$ 2.3 Hz
**H-3**	3.82	**1.50-1.43**, mostly obscured	4.84, ddd,<br>$J_{H-3,F}$ 49.5 Hz,<br>$J_{H-3,H-2}$ 8.5 Hz,<br>$J_{H-3,H-4}$ 3.5 Hz	3.65,<br>$J_{H-3,H-4}$ 3.2 Hz[11]	**1.47**, partially obscured, dd,<br>$J_{H-3,H-2}$ 11.6 Hz,<br>$J_{H-3,H-4}$ 2.6 Hz	4.60, ddd,<br>$J_{H-3,F}$ 52.3 Hz,<br>$J_{H-3,H-4}$ 9.2 Hz,<br>$J_{H-3,H-2}$ 3.5 Hz		**1.68**, partially obscured, app-t,<br>$J_{H-3,H-4}$ 10.8 Hz,<br>$J_{H-3,H-2}$ 11.6 Hz		**1.68**, partially obscured, dd,<br>$J_{H-3,H-4}$ 7.9 Hz,<br>$J_{H-3,H-2}$ 5.3 Hz
**H-4**	3.99,<br>$J_{H-4,H-5}$ <0.5 Hz[11]	**4.14-4.04**, partially obscured, broad s	4.36-4.19, m	3.92,<br>$J_{H-4,H-5}$ <0.5 Hz[11]	**4.12-4.08**, partially obscured, broad singlet	4.25, dt,<br>$J_{H-4,H-3}$ 3.4 Hz		**4.13**, dd,<br>$J_{H-4,H-5}$ 5.2 Hz,<br>$J_{H-4,H-3}$ 10.8 Hz		**4.40**, dd,<br>$J_{H-4,H-5}$ 4.1 Hz,<br>$J_{H-4,H-3}$ 8.3 Hz
**H-5**	4.09,<br>$J_{H-5,H-6}$ 6.2 Hz[11]	**~3.85-3.60**, in overlapping signals*		4.09	**3.78***	3.81, m		**3.75***		**~3.70***, in overlapping signals
**H-6**	3.78-3.72	**~3.69-3.61**, in overlapping signals	3.77, dd,<br>$J_{H-6,H-6'}$ 12.0 Hz,<br>$J_{H-6,H-5}$ 5.5 Hz	3.78-3.72	**~3.73-3.81**, in overlapping signals	3.81, m		**~3.75-3.60**, in overlapping signals		**~3.75-3.60**, in overlapping signals
**H-6'**	3.78-3.72		3.81, dd,<br>$J_{H-6',H-6}$ 12.0 Hz,<br>$J_{H-6',H-5}$ 7.0 Hz	3.78-3.72		3.81, m				

Legend: *Tentative assignment due to signal overlap. #Width of the ^{13}C-NMR signal. In green, C-3 signals are highlighted as their chemical shifts are very different from the C-3 signal chemical shifts for the D-galactose anomeric forms.

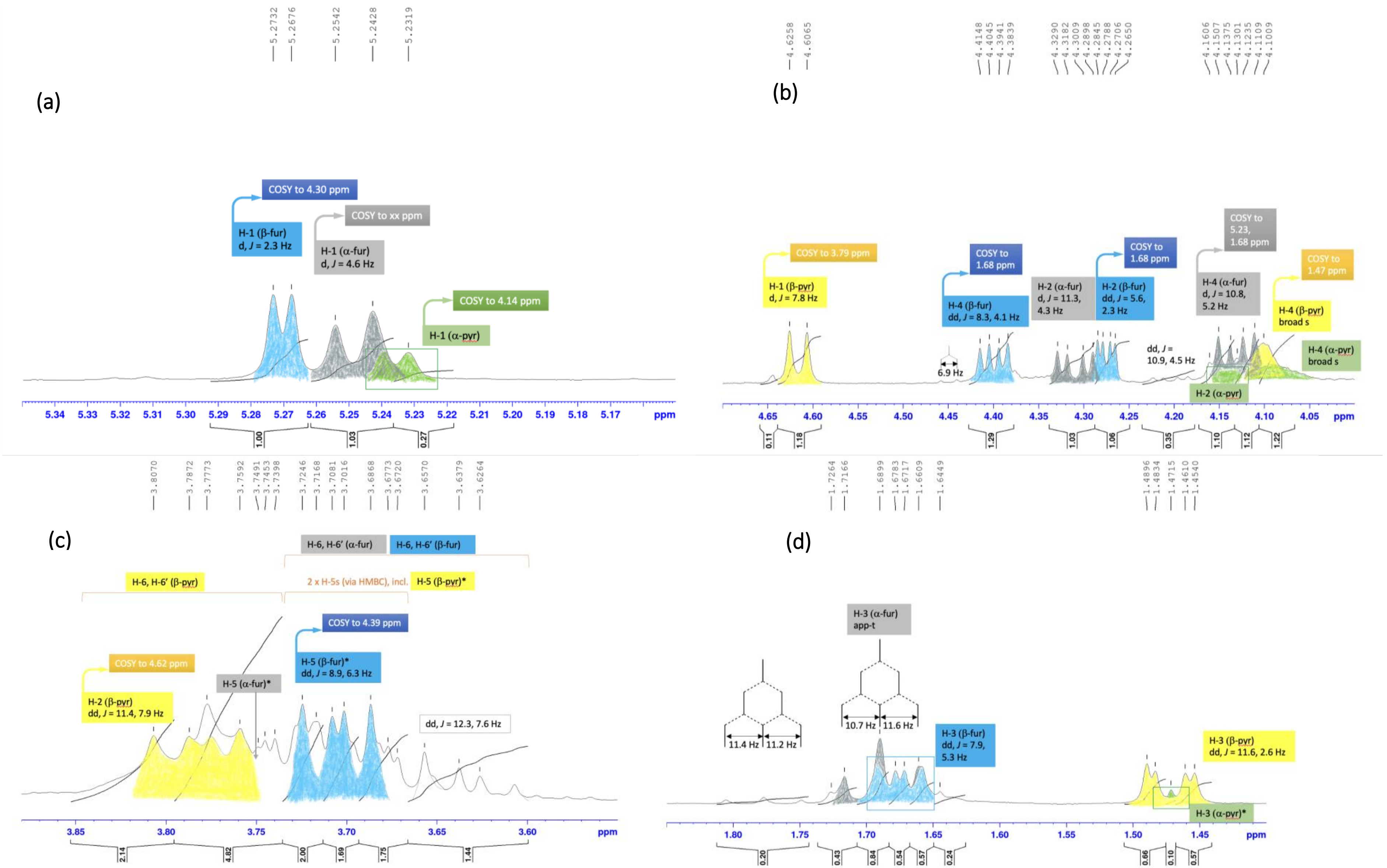

Figure S3. ^{1}H-NMR spectrum (400 MHz, D_2O) of the target compound 3-boronic-3-deoxy-D-galactose **2** (containing residual free DEA at 3.83 ppm and 3.20 ppm with colour-coded signals, highlighting the anomeric forms they belong to, with interpretation of the isolated signals and tentative interpretation of the overlapping ones. Namely, grey designates the α-fur form, indigo designates the β-fur form, green designates the α-pyr form, yellow designates the β-pyr form. (a) section 5.35 ppm to 5.16 ppm; (b) section 4.70 ppm to 4.00 ppm; (c) section 3.87 ppm to 3.55 ppm; (d) section 1.85 ppm to 1.40 ppm. Highlighted are also the principal COSY correlations to hydrogens within the same spin systems.

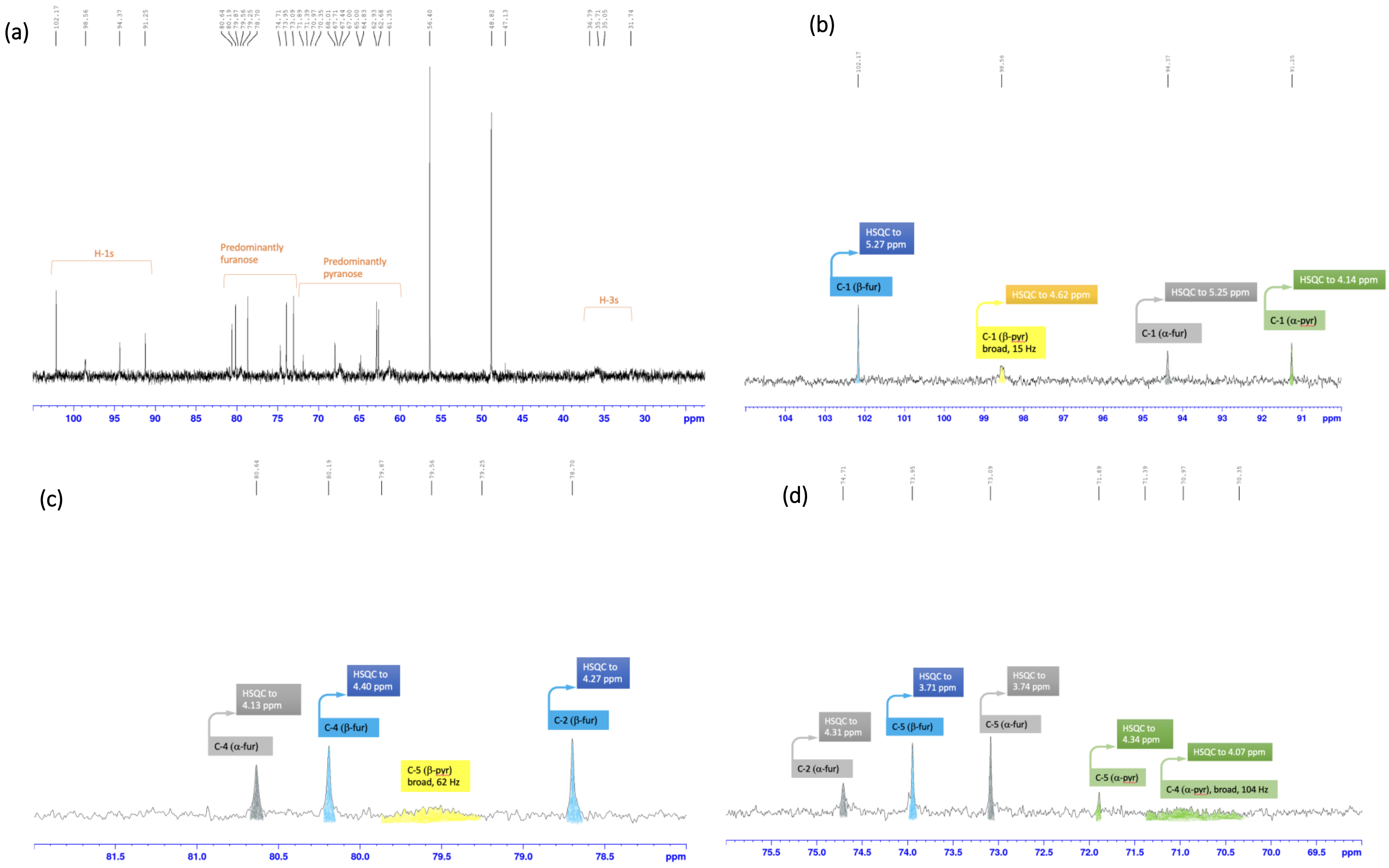

(a)
H-1s
Predominantly furanose
Predominantly pyranose
H-3s
ppm
(b)
HSQC to 5.27 ppm
C-1 (β-fur)
HSQC to 4.62 ppm
C-1 (β-pyr) broad, 15 Hz
HSQC to 5.25 ppm
C-1 (α-fur)
HSQC to 4.14 ppm
C-1 (α-pyr)
ppm
(c)
HSQC to 4.13 ppm
C-4 (α-fur)
HSQC to 4.40 ppm
C-4 (β-fur)
C-5 (β-pyr) broad, 62 Hz
HSQC to 4.27 ppm
C-2 (β-fur)
ppm
(d)
HSQC to 4.31 ppm
C-2 (α-fur)
HSQC to 3.71 ppm
C-5 (β-fur)
HSQC to 3.74 ppm
C-5 (α-fur)
HSQC to 4.34 ppm
C-5 (α-pyr)
HSQC to 4.07 ppm
C-4 (α-pyr), broad, 104 Hz
ppm

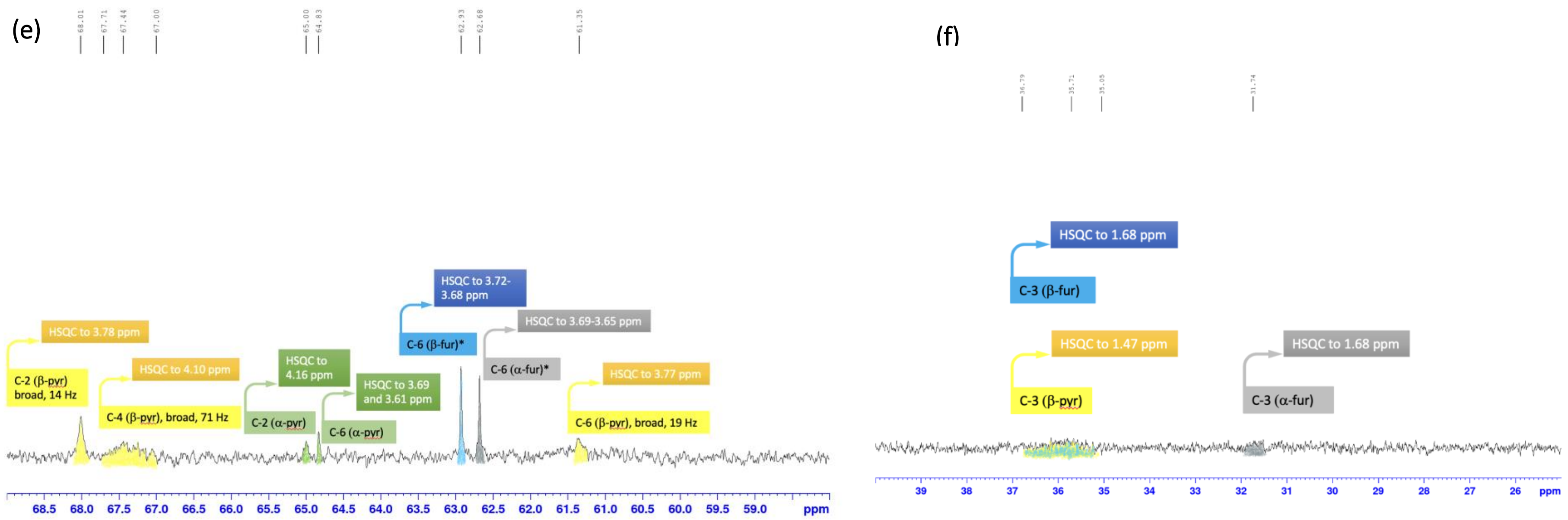

Figure S4. ^{13}C-NMR spectrum (100 MHz, D_2O) of the target compound 3-boronic-3-deoxy-D-galactose **2** (containing residual free DEA at 3.83 ppm and 3.20 ppm [see Supplementary Information]) with colour-coded signals, highlighting the anomeric forms they belong to. Namely, grey designates the α-fur form, indigo designates the β-fur form, green designates the α-pyr form, yellow designates the β-pyr form. (a) the whole spectrum with overview of chemical shift regions where signals are found; (b) section 105.0 ppm to 90.0 ppm; (c) section 82.0 ppm to 78.0 ppm; (d) section 76.0 ppm to 69.0 ppm; (e) section 69.0 ppm to 58.0 ppm; (f) section 40 ppm to 25 ppm. Highlighted are also the principal HSQC correlations to hydrogens within the same spin systems.

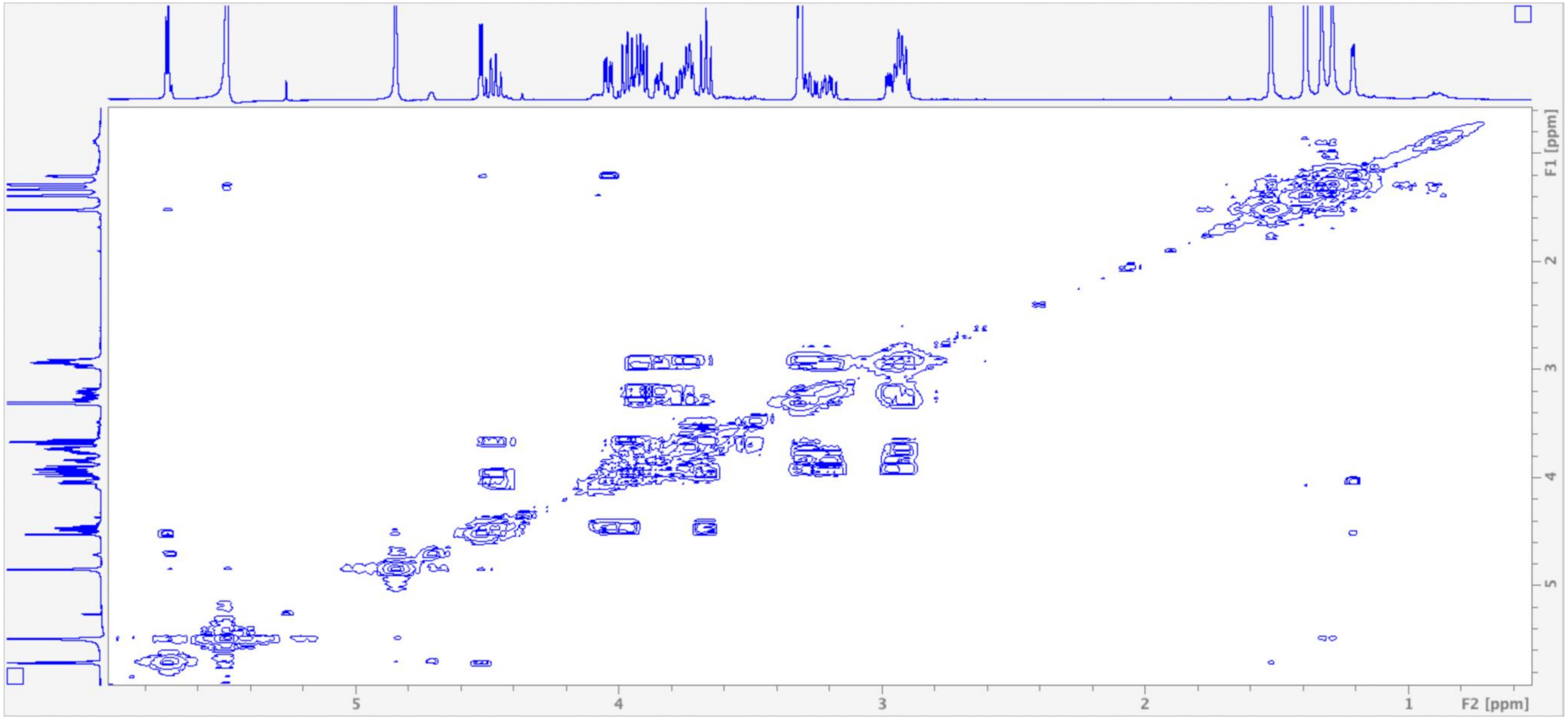

(a)

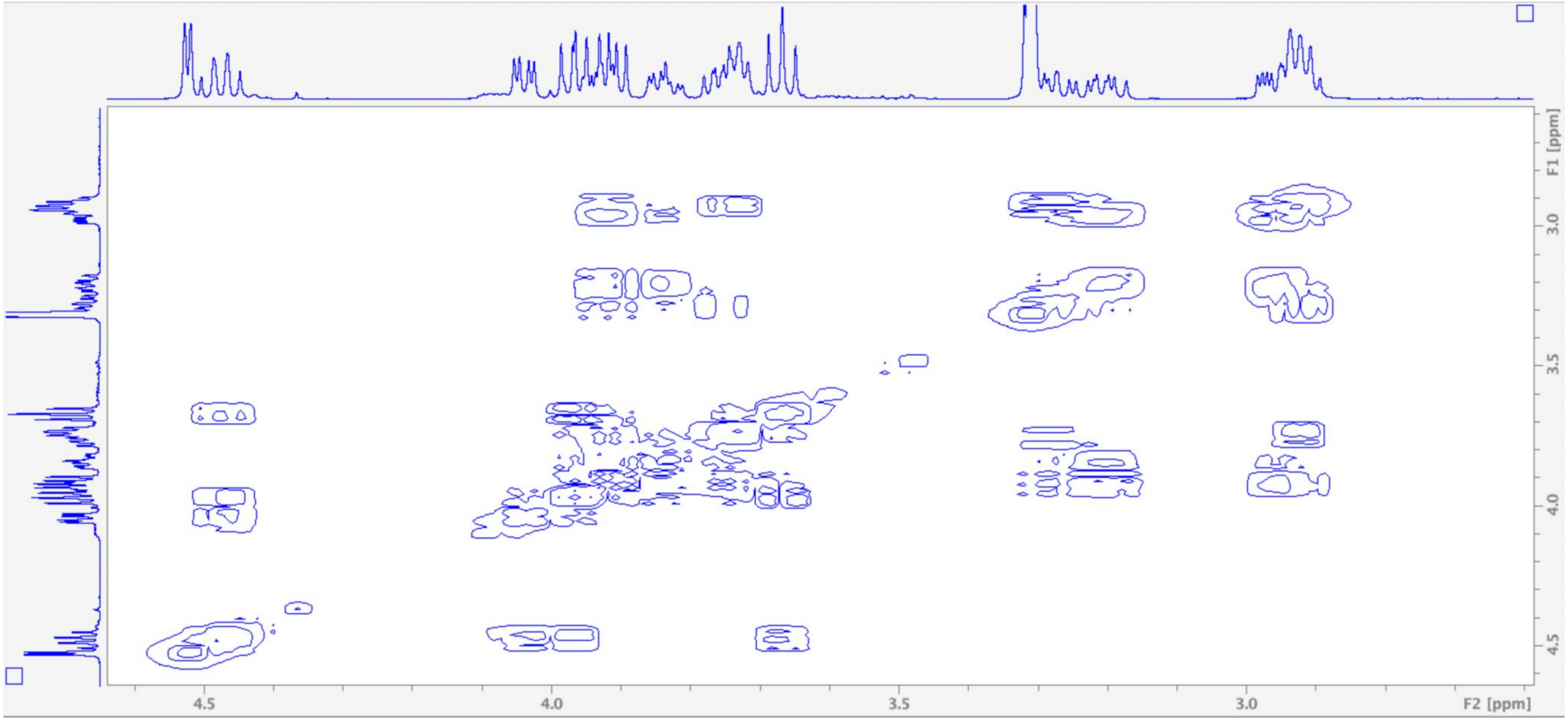

(b)

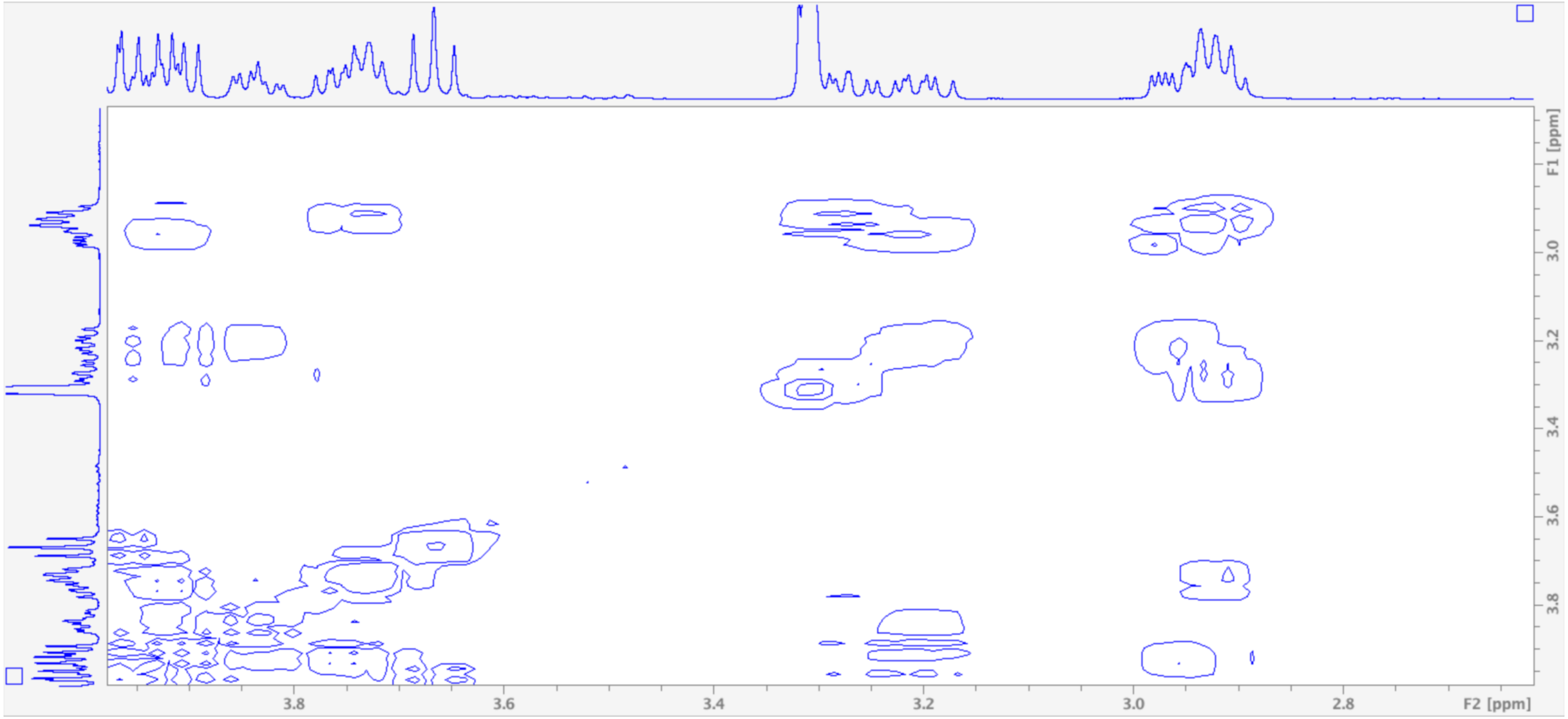

(c)

Figures S5. COSY Spectra (a), (b) and (c) (MeOD) of 3-deoxy-3-boronodiethanolamine-1,2:5,6-di-*O*-isopropylidene-α-D-galactofuranose **1**.

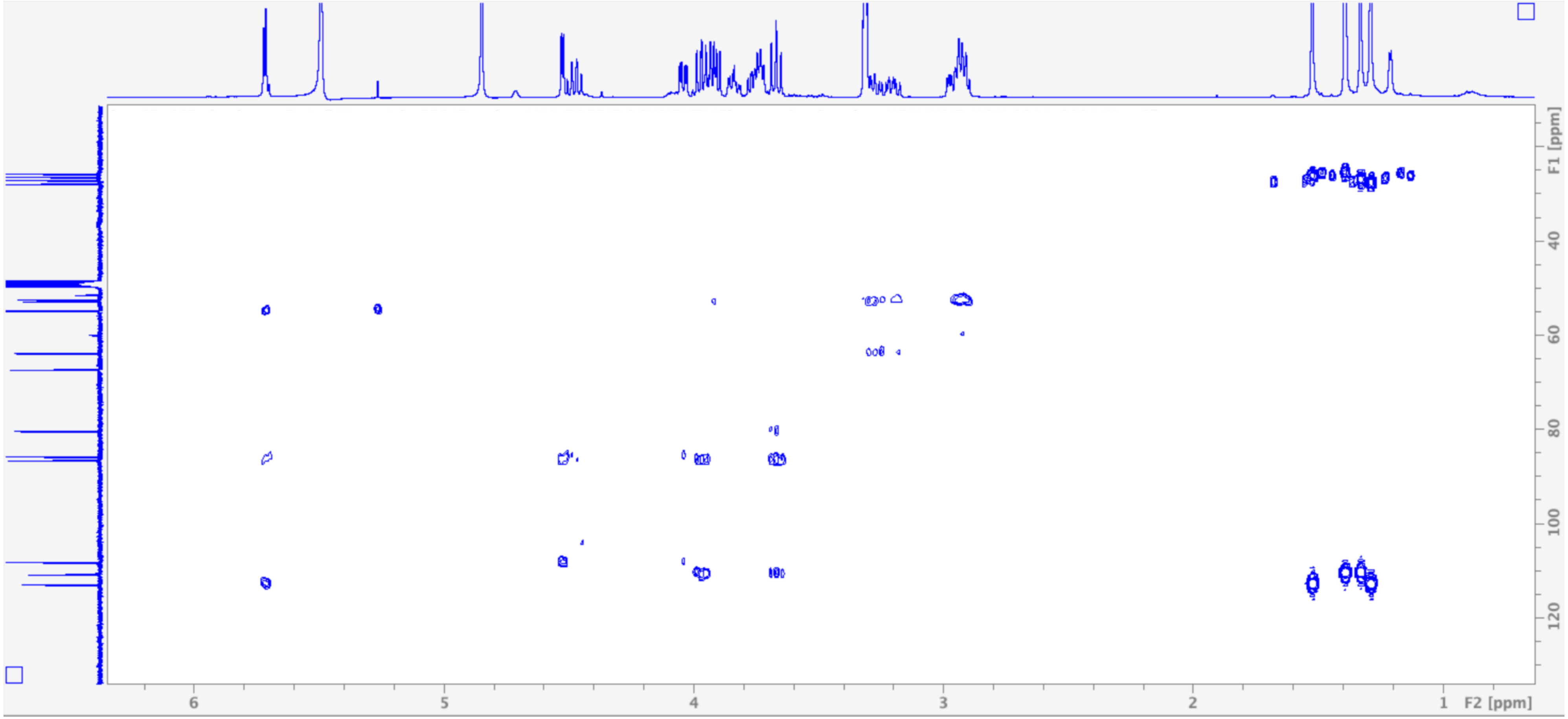

Figure S6. HMBC Spectrum (MeOD) of 3-deoxy-3-boronodiethanolamine-1,2:5,6-di-*O*-isopropylidene-α-D-galactofuranose **1**.

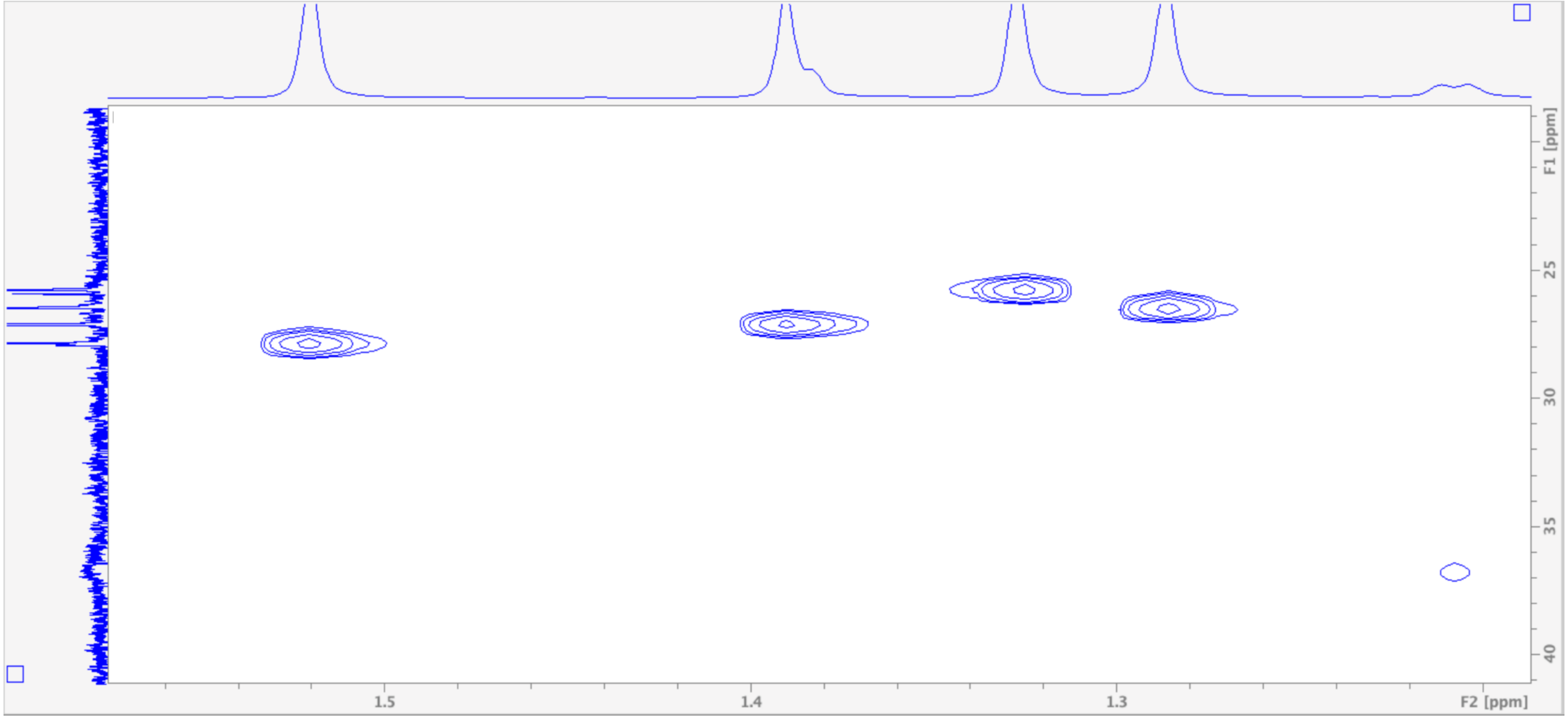

(a)

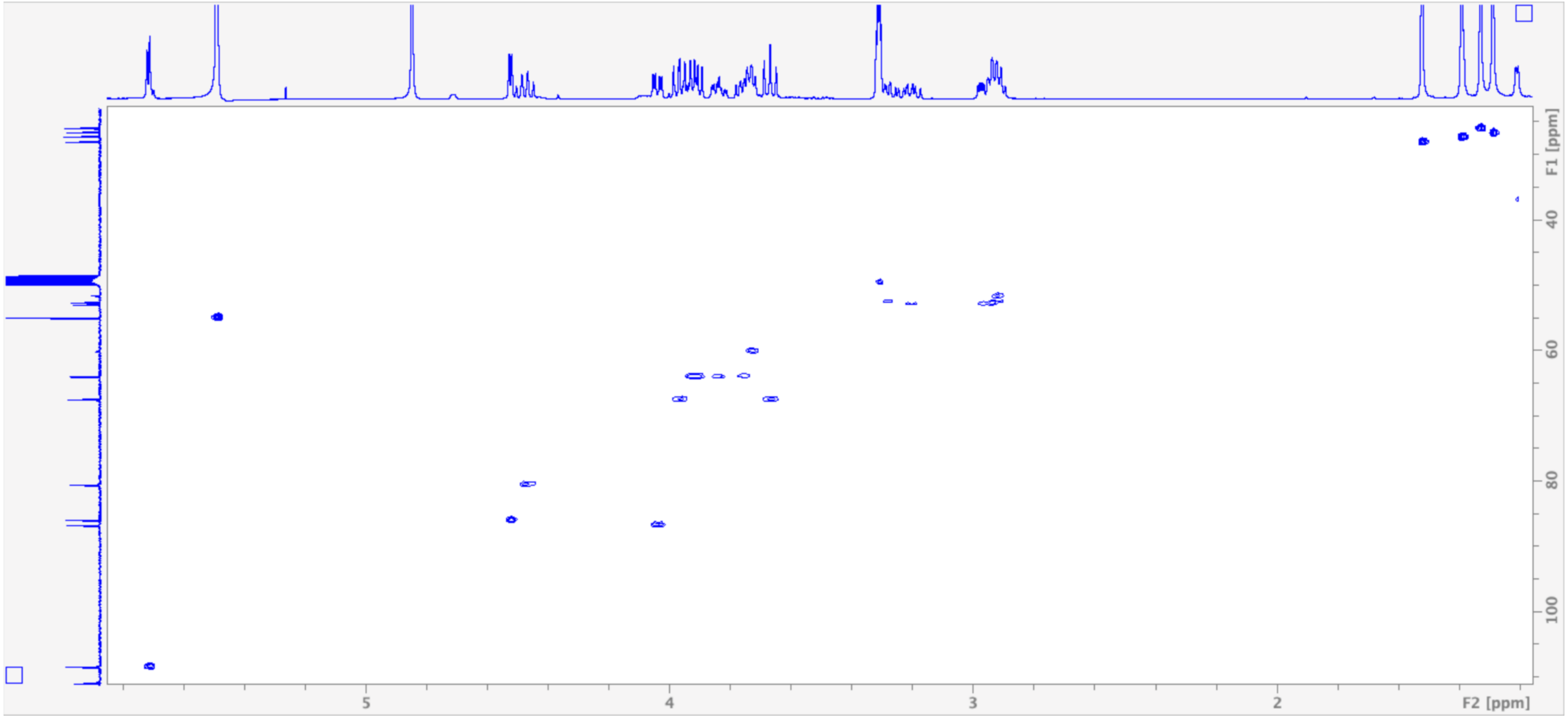

(b)

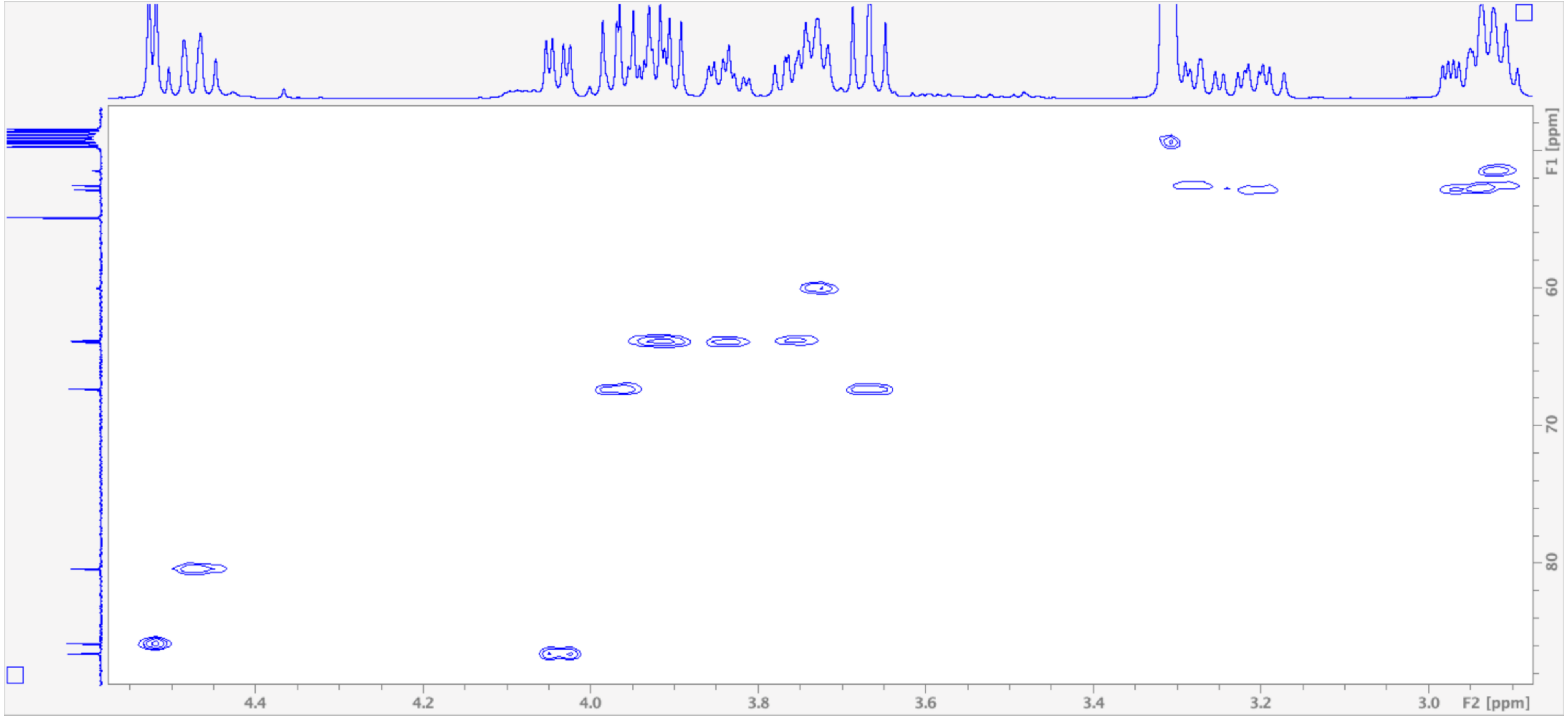

(c)

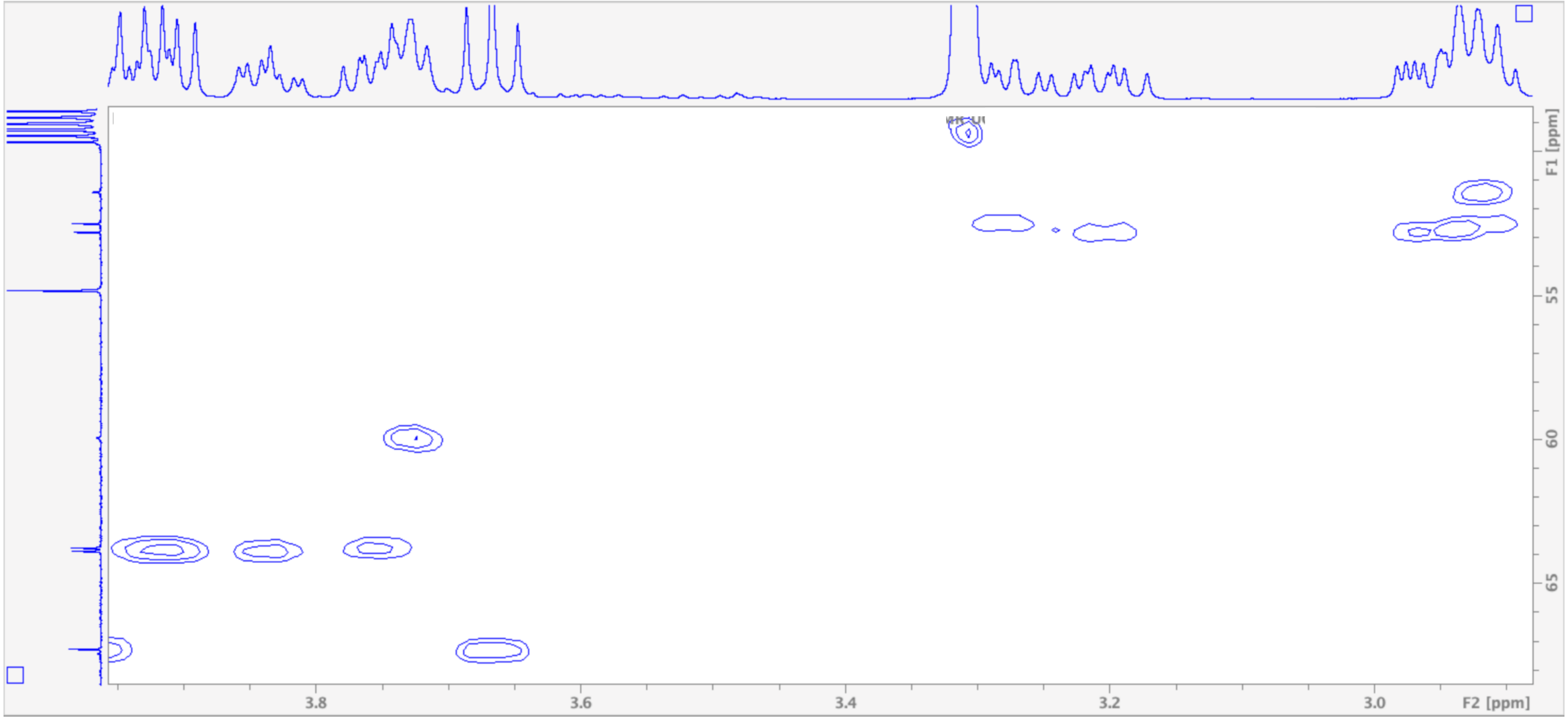

(d)

Figures S7. HSQC Spectra (a), (b), (c) and (d) (MeOD) of 3-deoxy-3-boronodiethanolamine-1,2:5,6-di-*O*-isopropylidene-α-D-galactofuranose **1**.

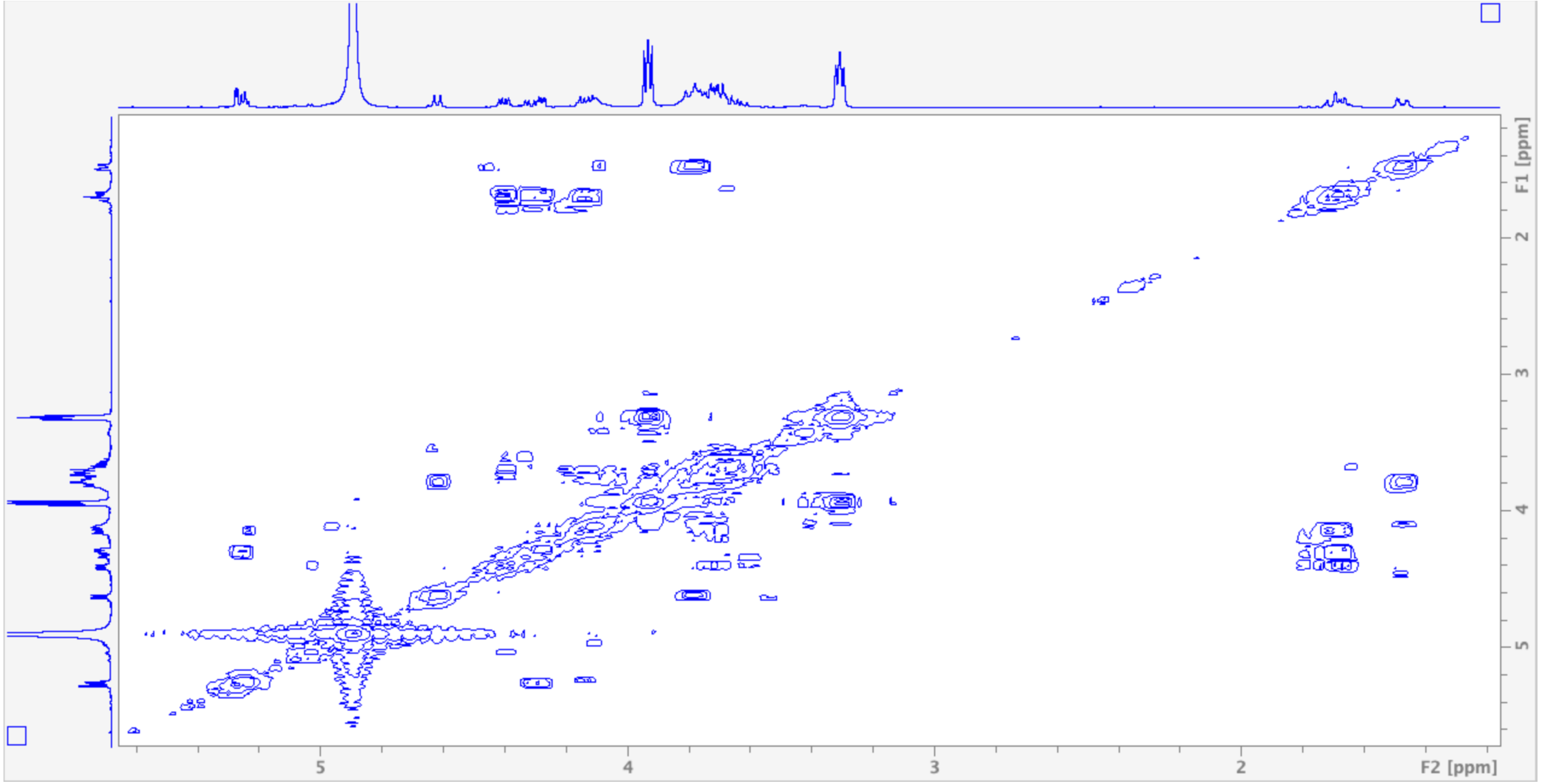

(a)

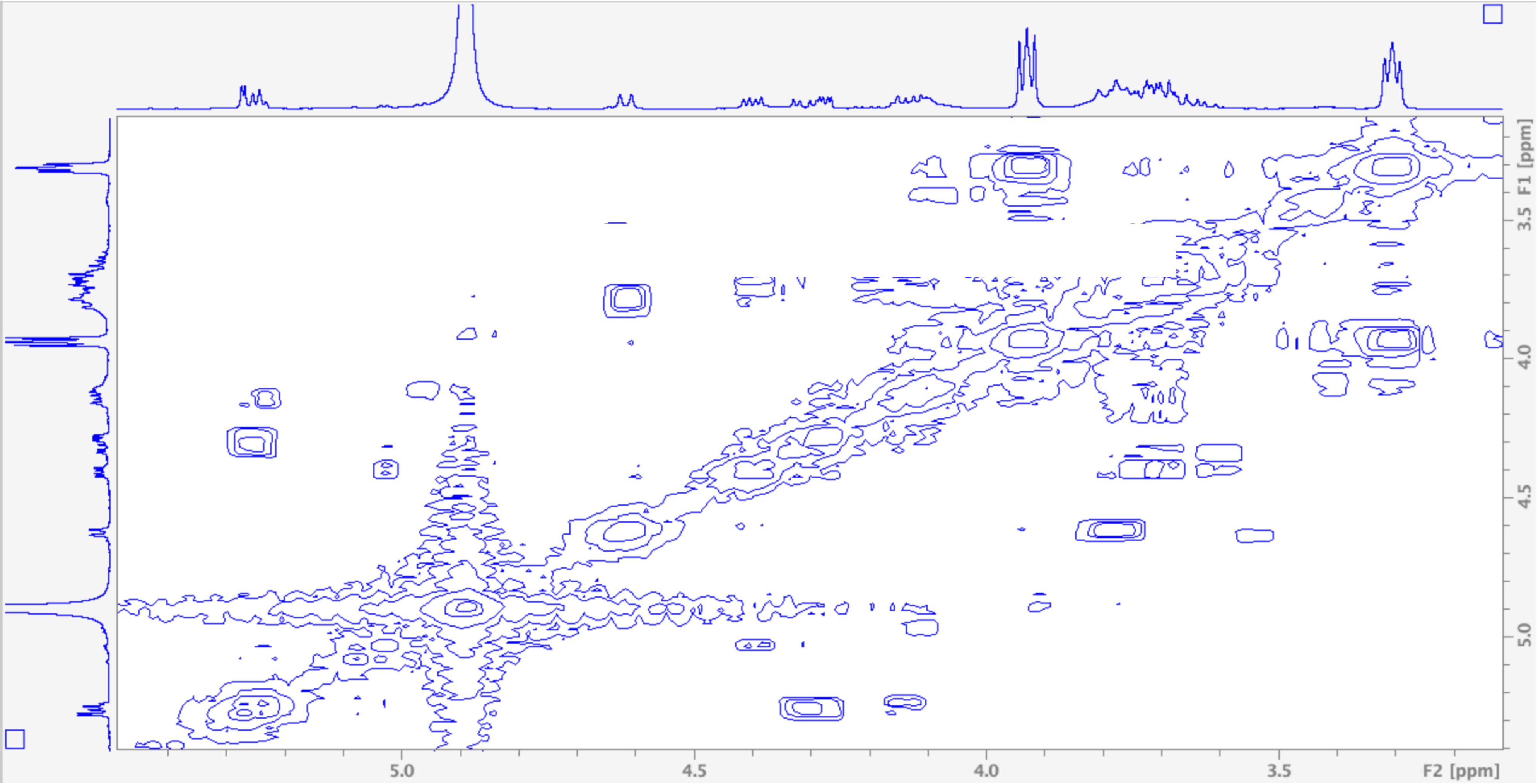

(b)

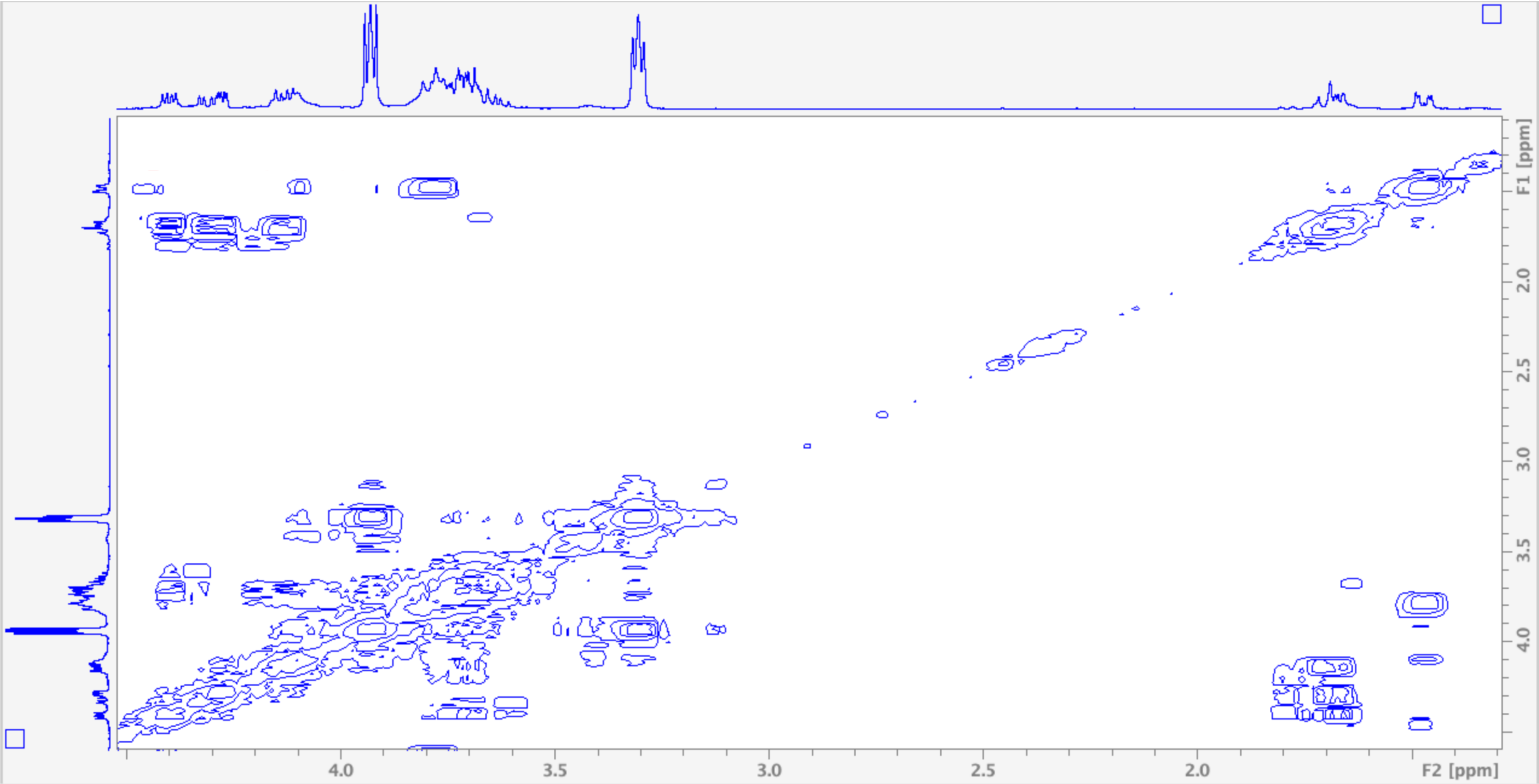

(c)

Figures S8. COSY Spectra (a), (b) and (c) (D_2O) of the target compound 3-boronic-3-deoxy-D-galactose **2**.

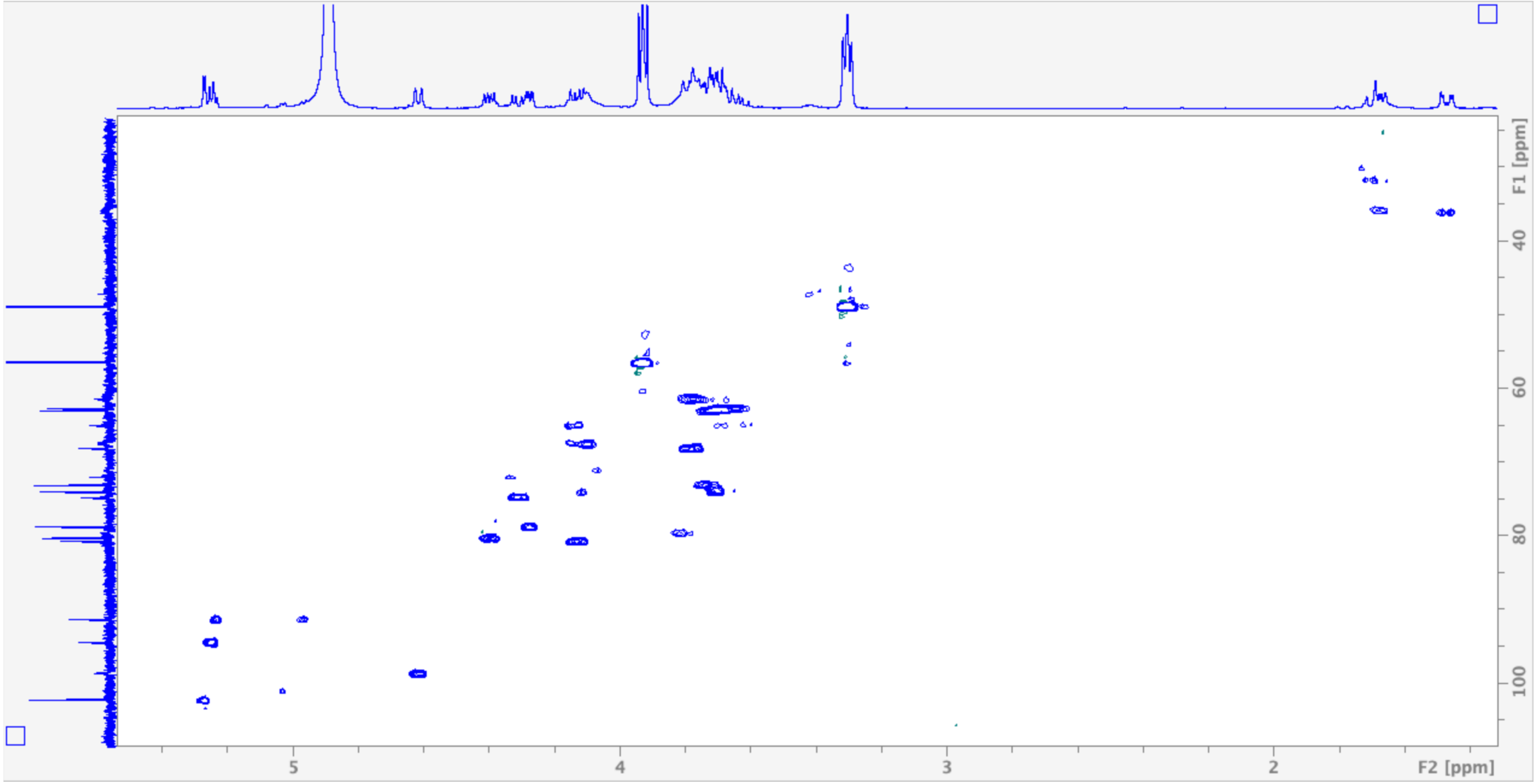

(a)

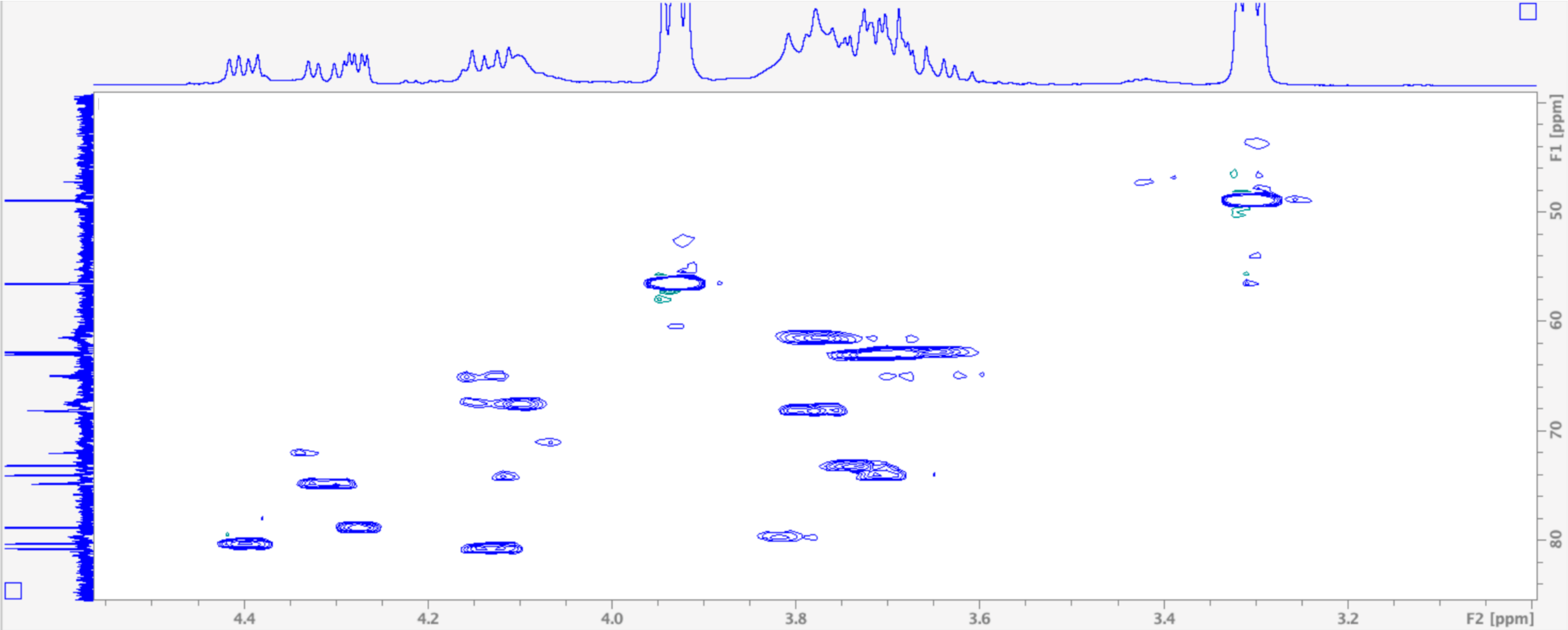

(b)

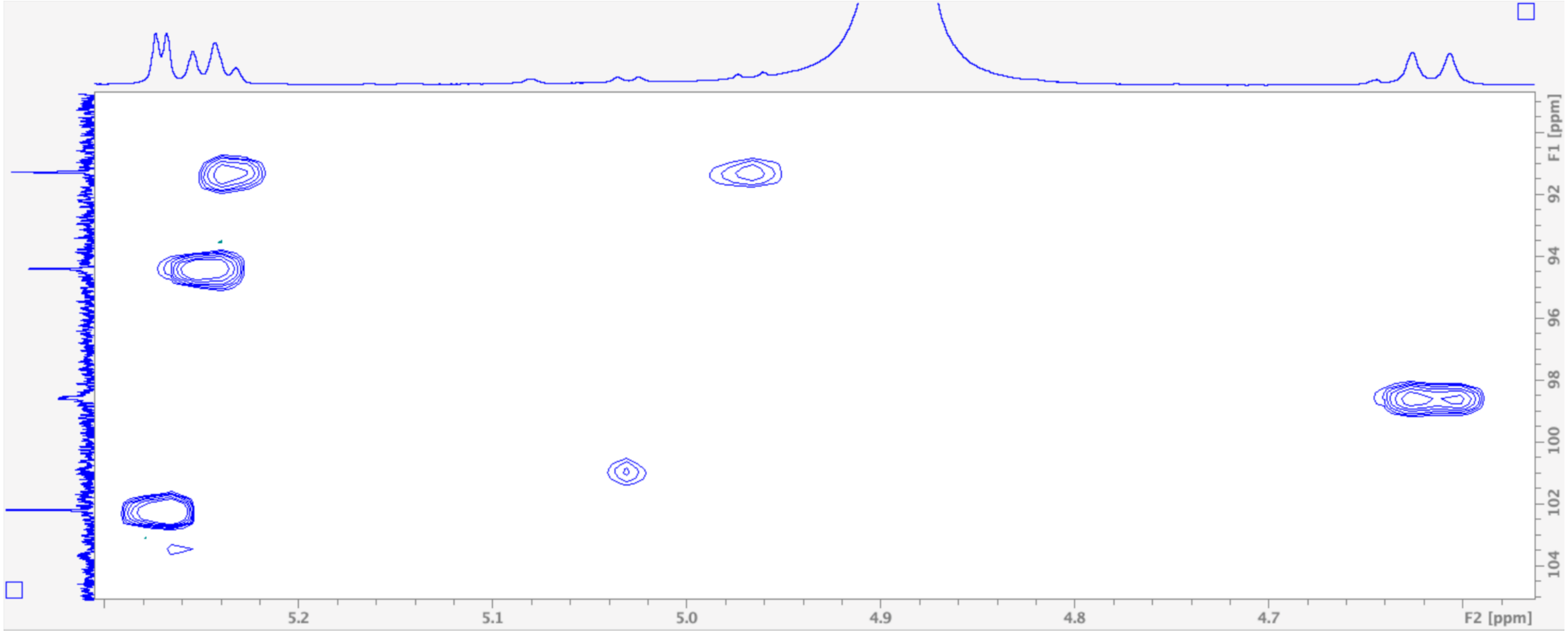

(c)

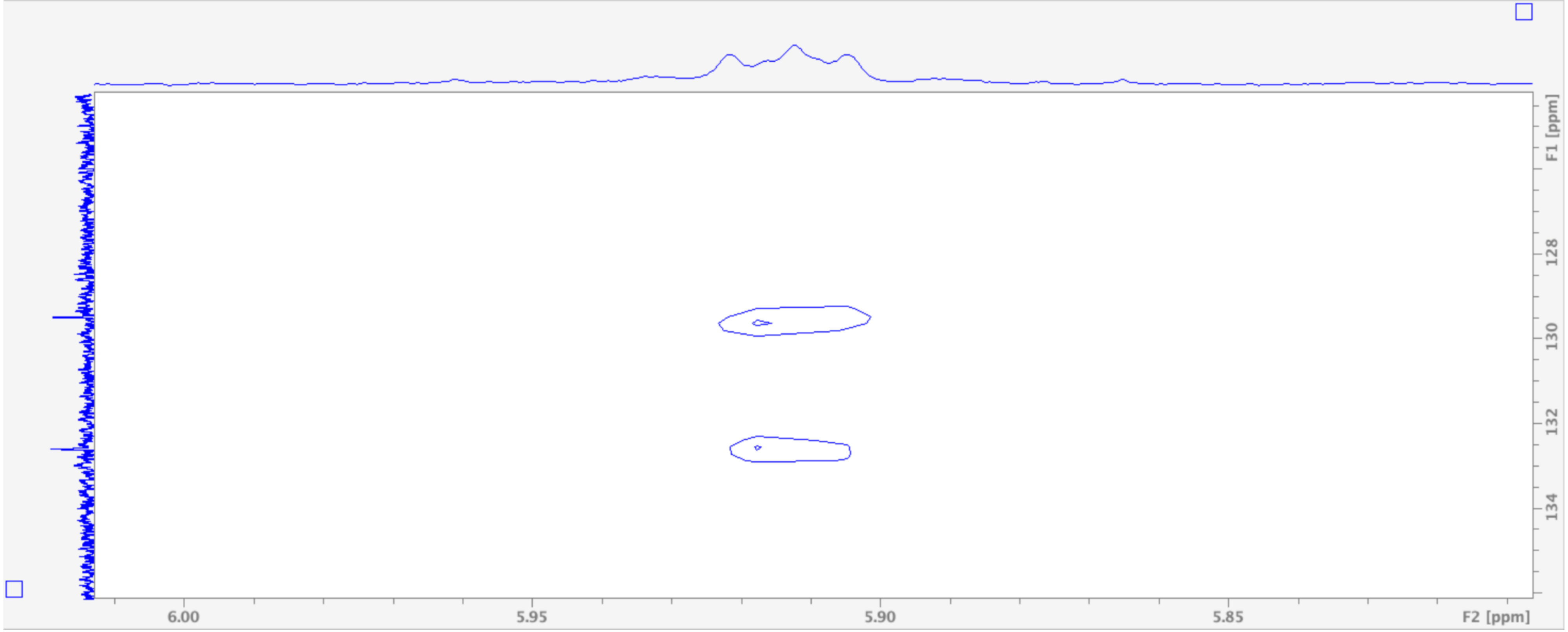

(d)

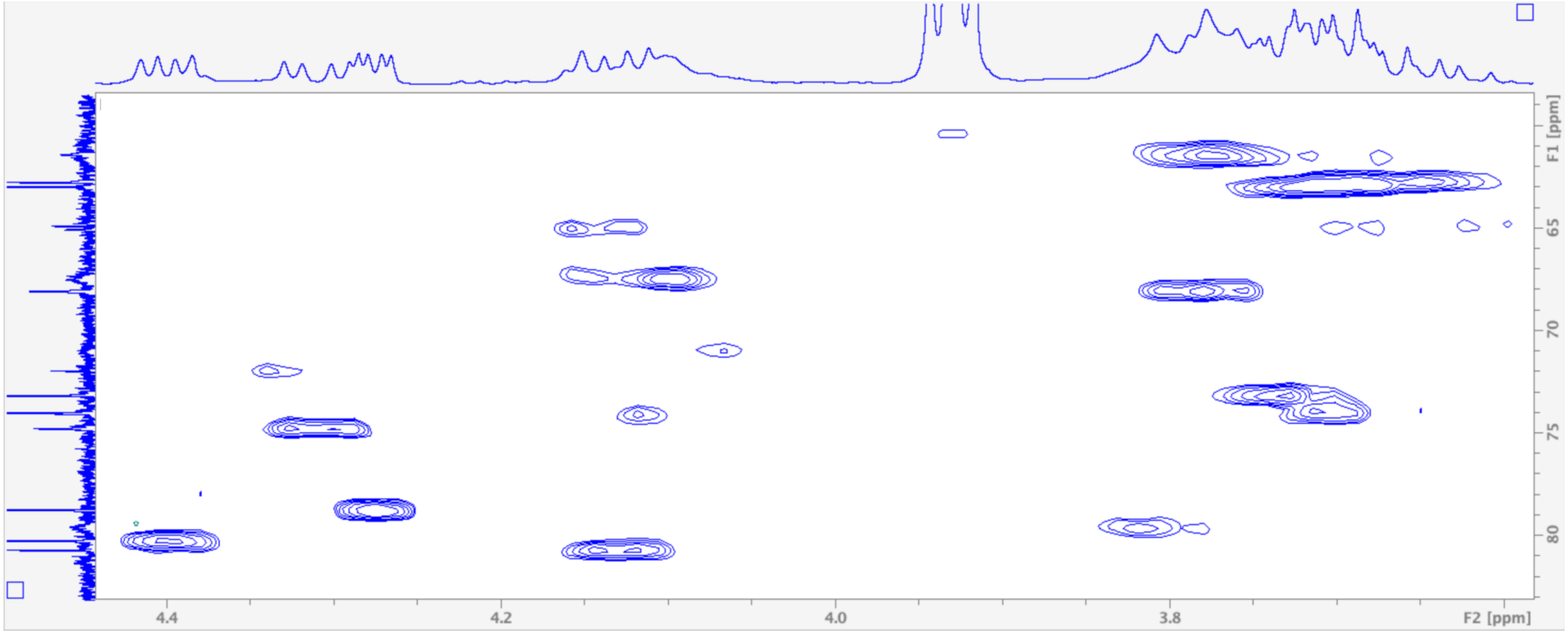

(e)

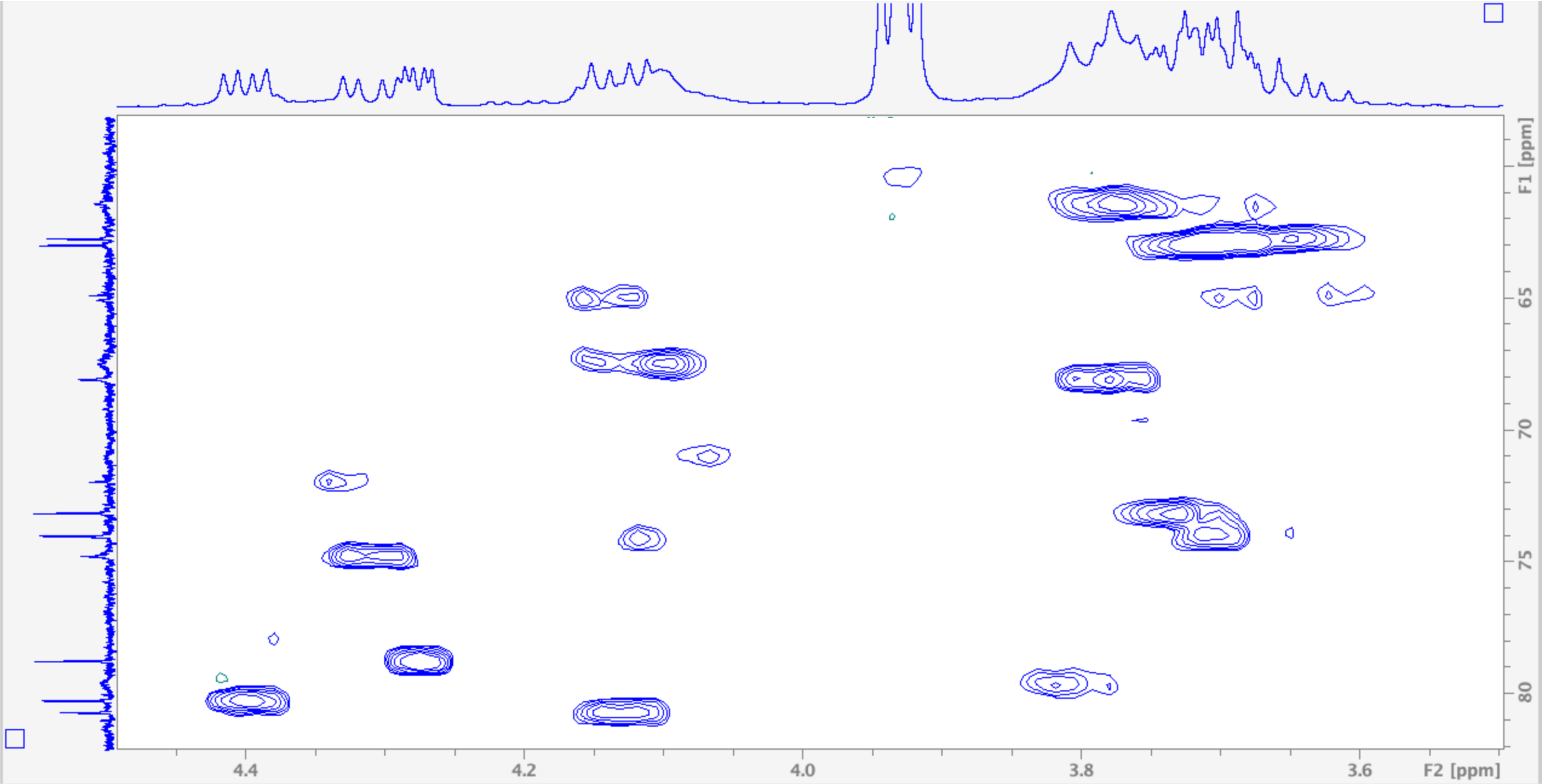

(f)

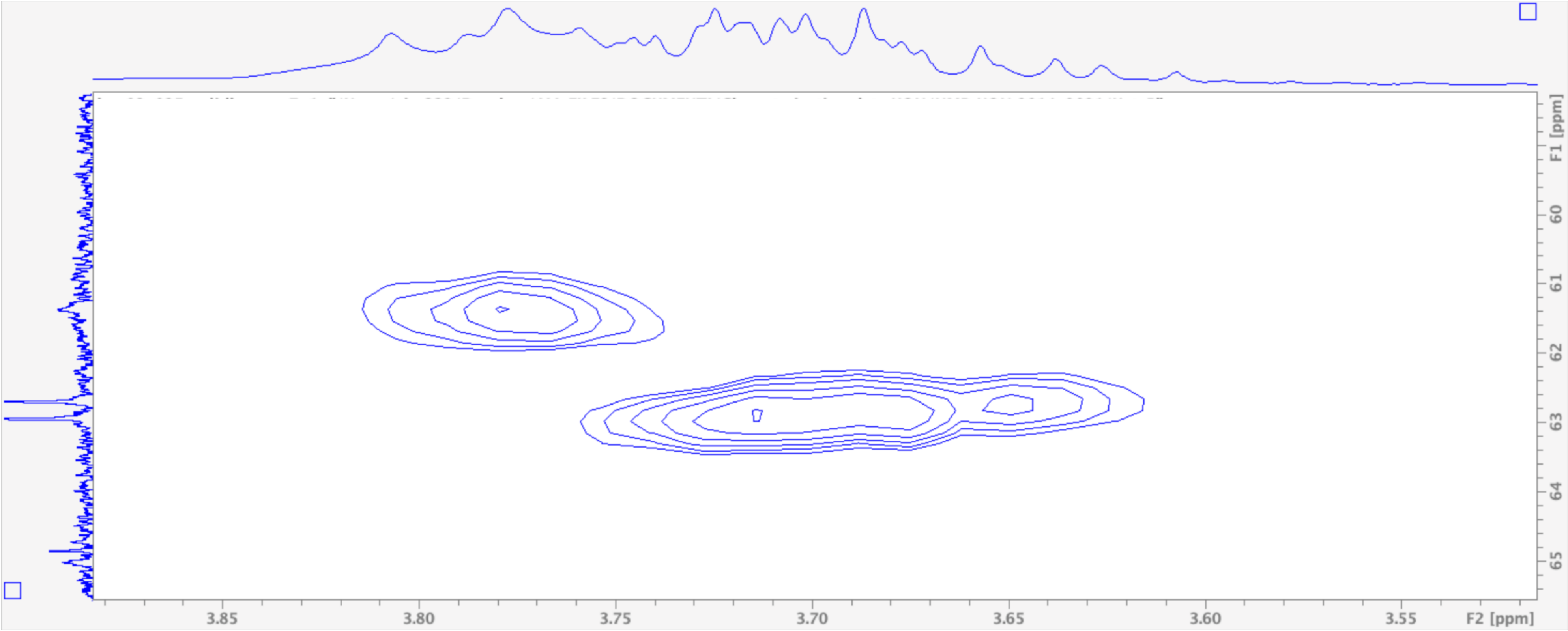

(g)

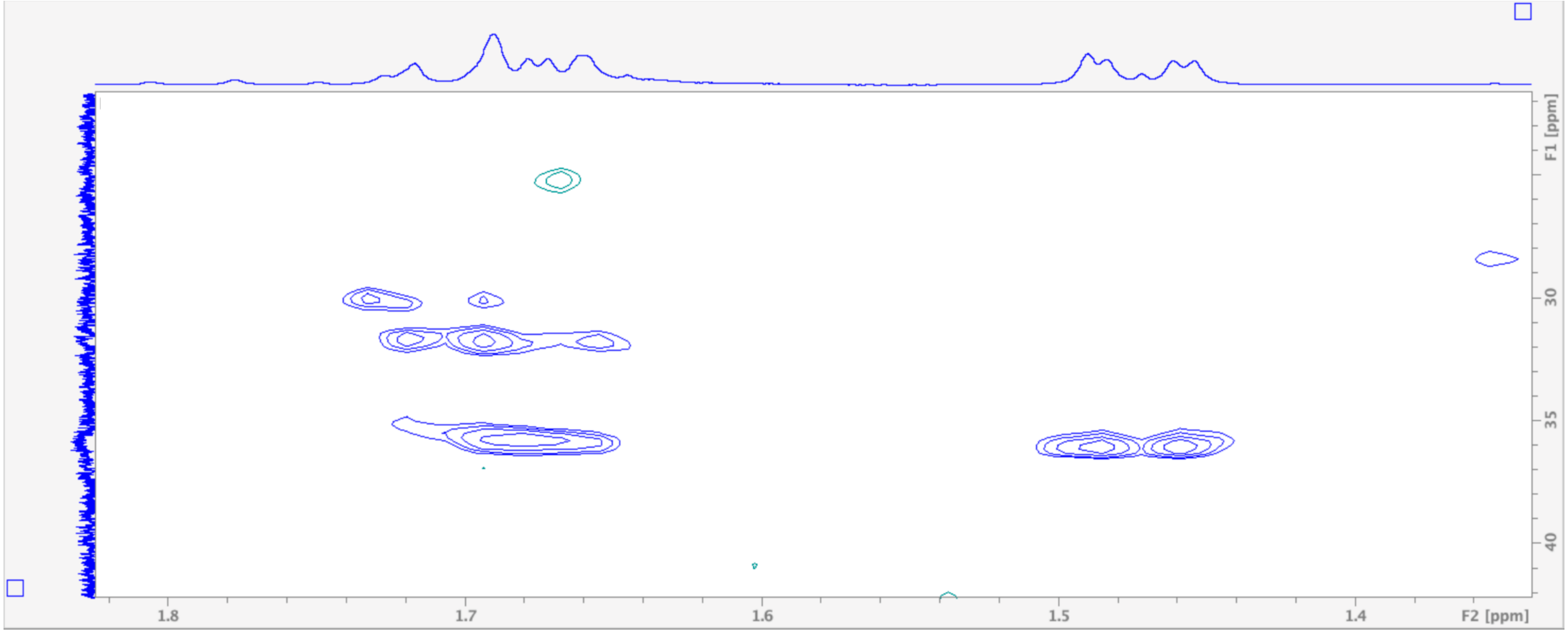

(h)

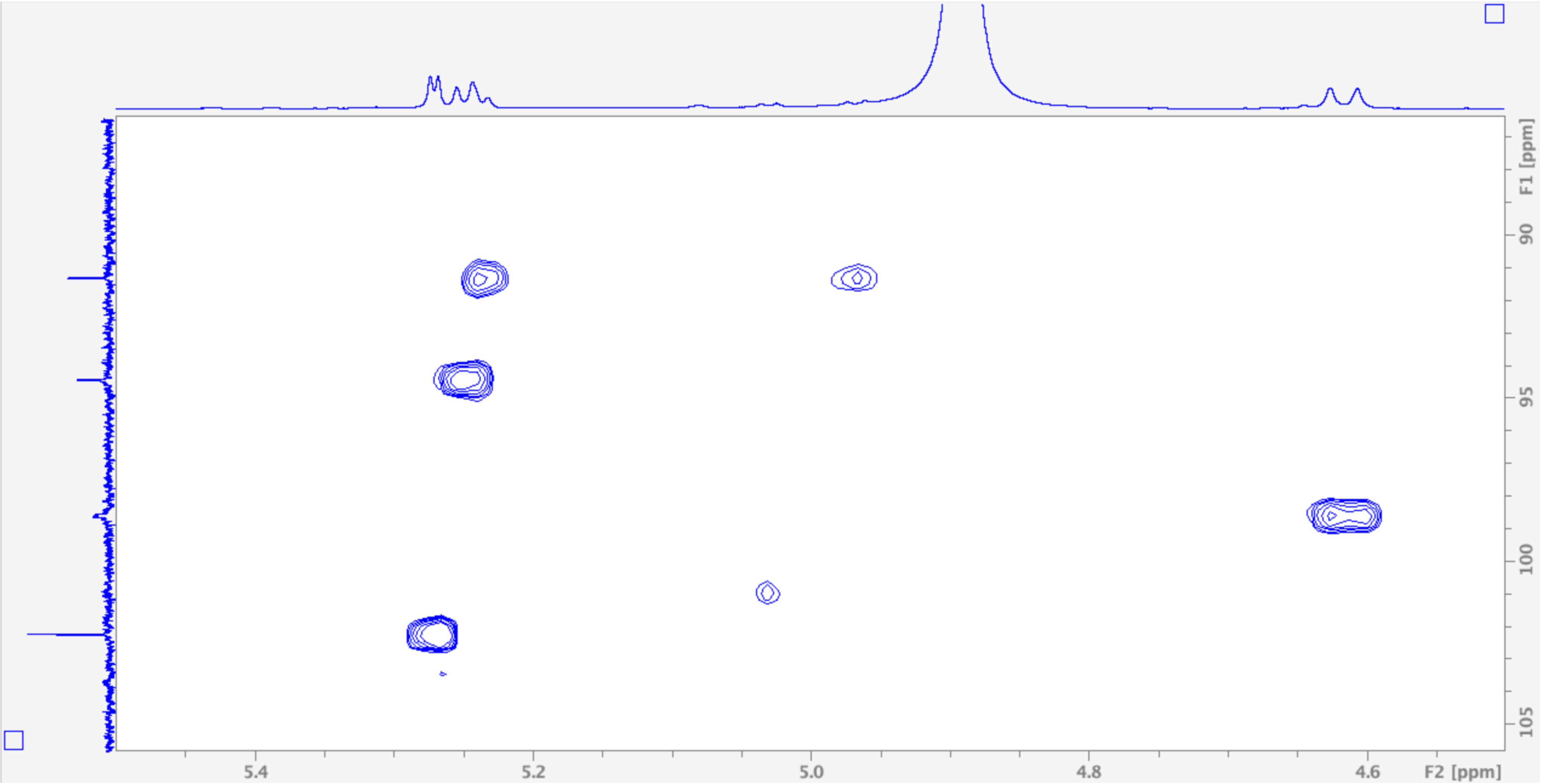

(i)

Figures S9. HSQC Spectra (a), (b), (c), (d), (e), (f), (g), (h) and (i) (D_2O) of the target compound 3-boronic-3-deoxy-D-galactose **2**.

References

1. Campkin, D.M.; Shimadate, Y.; Bartholomew, B.; Bernhardt, P.V.; Nash, R.J.; Sakoff, J.A.; Kato, A.; Simone, M. Borylated 2,3,4,5-Tetrachlorophthalimide and Their 2,3,4,5-Tetrachlorobenzamide Analogues: Synthesis, Their Glycosidase Inhibition and Anticancer Properties in View to Boron Neutron Capture Therapy. *Molecules* **2022**, *27*, 3447-3475, doi:https://doi.org/10.3390/molecules27113447.
2. Legge, W.J.; Shimadate, Y.; Sakoff, J.; Houston, T.A.; Kato, A.; Bernhardt, P.V.; Simone, M. Borylated methyl cinnamates: Green synthesis, characterization, crystallographic analysis and biological activities in glycosidase inhibition and in cancer cells lines. *Beilstein Arch.* **2021**, 20214, doi:https://doi.org/10.3762/bxiv.2021.4.v1.
3. Wrackmeyer, B. Nuclear magnetic resonance spectroscopy of boron compounds containing two-, three-and four-coordinate boron. *Ann. Rep. NMR Spectr.* **1988**, *20*, 61-203, doi:https://doi.org/10.1016/S0066-4103(08)60170-2.
4. Weiss, J.W.; Bryce, D.L. A solid-state 11B NMR and computational study of boron electric field gradient and chemical shift tensors in boronic acids and boronic esters. *J. Phys. Chem. A* **2010**, *114*, 5119-5131.
5. Zhuo, J.C.; Soloway, A.H.; Beeson, J.C.; Ji, W.; Barnum, B.A.; Rong, F.G.; Tjarks, W.; Jordan, G.T.; Liu, J.; Shore, S.G. Boron-containing heterocycles: syntheses, structures, and properties of benzoborauracils and a benzoborauracil nucleoside. *J. Org. Chem.* **1999**, *64*, 9566-9574.
6. Raju, R.; Castillo, B.F.; Richardson, S.K.; Thakur, M.; Severins, R.; Kronenberg, M.; Howell, A.R. Synthesis and evaluation of 3’’- and 4’’-deoxy and -fluoro analogs of the immunostimulatory glycolipid, KRN7000. *Bioorg. Med. Chem. Lett.* **2009**, *19*, 4122-4125, doi:10.1016/j.bmcl.2009.06.005.
7. Zhang, Q.; Liu, H.-W. Mechanistic Investigation of UDP-Galactopyranose Mutase from Escherichia coli Using 2- and 3-Fluorinated UDP-Galactofuranose as Probes. *J. Am. Chem. Soc.* **2001**, *123*, 6756-6766, doi:https://doi.org/10.1021/ja010473l.
8. Bock, K.; Pedersen, C. Carbon-13 Nuclear Magnetic Resonance Spectroscopy of Monosaccharides. *Adv. Carb. Chem. Biochem.* **1983**, *41*, 27-66, doi:https://doi.org/10.1016/S0065-2318(08)60055-4
9. Voelter, W.; Breitmaier, E.; Rathbone, E.B.; Stephen, A.M. The influence of methylation on 13C chemical shifts of galactose derivatives. *Tetrahedron* **1973**, *29*, 3845-3848, doi:https://doi.org/10.1016/0040-4020(73)80204-2.
10. Zhu, Y.; Zajicek, J.; Serianni, A.S. Acyclic Forms of [1-13C]Aldohexoses in Aqueous Solution: Quantitation by 13C NMR and Deuterium Isotope Effects on Tautomeric Equilibria. *J. Org. Chem.* **2001**, *66*, 6244-6251, doi:https://doi.org/10.1021/jo010541m.
11. Kunihiko, I. NMR Spectra of Some Monosaccharides of Galactopyranose Series in Deuterium Oxide. *Agr. Biol. Chem.* **1971**, *35*, 1816-1818, doi:https://doi.org/10.1080/00021369.1971.10860150.
12. Lemieux, R.U.; Stevens, J.D. The proton magnetic resonance spectra and tautomeric equilibria of aldoses in deuterium oxide. *Can. J. Chem.* **1966**, *44*, 249-262, doi:https://doi.org/10.1139/v66-037.
13. Angyal, S.J.; Pickles, V.A. Equilibria between pyranoses and furanoses. *Aust. J. Chem.* **1972**, *25*, 1695-1710.
